# Dravet syndrome-associated mutations in *GABRA1*, *GABRB2* and *GABRG2* define the genetic landscape of defects of GABA_A_ receptors

**DOI:** 10.1093/braincomms/fcab033

**Published:** 2021-03-11

**Authors:** Ciria C Hernandez, XiaoJuan Tian, Ningning Hu, Wangzhen Shen, Mackenzie A Catron, Ying Yang, Jiaoyang Chen, Yuwu Jiang, Yuehua Zhang, Robert L Macdonald

**Affiliations:** 1 Life Sciences Institute, University of Michigan, Ann Arbor, MI 48198, USA; 2 Department of Neurology, Vanderbilt University Medical Center, Nashville, TN 37240, USA; 3 Department of Pediatrics and Pediatric Epilepsy Center, Peking University First Hospital, Beijing 100034, China; 4 Department of Neurology, Beijing Children’s Hospital, Capital Medical University, National Center for Children’s Health, Beijing 100045, China; 5 Neuroscience Graduate Program, Vanderbilt University, Nashville, TN 37240, USA; 6 Center of Epilepsy, Beijing Institute for Brain Disorders, Beijing 100069, China

**Keywords:** Dravet syndrome-associated mutations, *GABRA1*, *GABRB2*, *GABRG2*, PIP_2_

## Abstract

Dravet syndrome is a rare, catastrophic epileptic encephalopathy that begins in the first year of life, usually with febrile or afebrile hemiclonic or generalized tonic–clonic seizures followed by status epilepticus. *De novo* variants in genes that mediate synaptic transmission such as *SCN1A* and *PCDH19* are often associated with Dravet syndrome. Recently, GABA_A_ receptor subunit genes (*GABRs*) encoding α1 (*GABRA1*), β3 (*GABRB3*) and γ2 (*GABRG2*), but not β2 (*GABRB2*) or β1 (*GABRB1*), subunits are frequently associated with Dravet syndrome or Dravet syndrome-like phenotype. We performed next generation sequencing on 870 patients with Dravet syndrome and identified nine variants in three different *GABRs*. Interestingly, the variants were all in genes encoding the most common GABA_A_ receptor, the α1β2γ2 receptor. Mutations in *GABRA1* (c.644T>C, p. L215P; c.640C>T, p. R214C; c.859G>A; V287I; c.641G>A, p. R214H) and *GABRG2* (c.269C>G, p. T90R; c.1025C>T, p. P342L) presented as *de novo* cases, while in *GABRB2* two variants were *de novo* (c.992T>C, p. F331S; c.542A>T, p. Y181F) and one was autosomal dominant and inherited from the maternal side (c.990_992del, p.330_331del). We characterized the effects of these *GABR* variants on GABA_A_ receptor biogenesis and channel function. We found that defects in receptor gating were the common deficiency of *GABRA1* and *GABRB2* Dravet syndrome variants, while mainly trafficking defects were found with the *GABRG2* (c.269C>G, p. T90R) variant. It seems that variants in α1 and β2 subunits are less tolerated than in γ2 subunits, since variant α1 and β2 subunits express well but were functionally deficient. This suggests that all of these *GABR* variants are all targeting *GABR* genes that encode the assembled α1β2γ2 receptor, and regardless of which of the three subunits are mutated, variants in genes coding for α1, β2 and γ2 receptor subunits make them candidate causative genes in the pathogenesis of Dravet syndrome.

## Teaser

Next-generation sequencing on 870 patients with Dravet syndrome identified nine variants in *GABRA1*, *GABRB2* and *GABRG2* genes, which encode the most common α1β2γ2 GABA_A_ receptor in the CNS. Hernandez et al. report that mutations in multiple genes (*GABRA1*, *GABRB2* and *GABRG2*) have a common target (α1β2γ2) to cause Dravet syndrome.

## Graphical Abstract

**Graphical Abstract:**
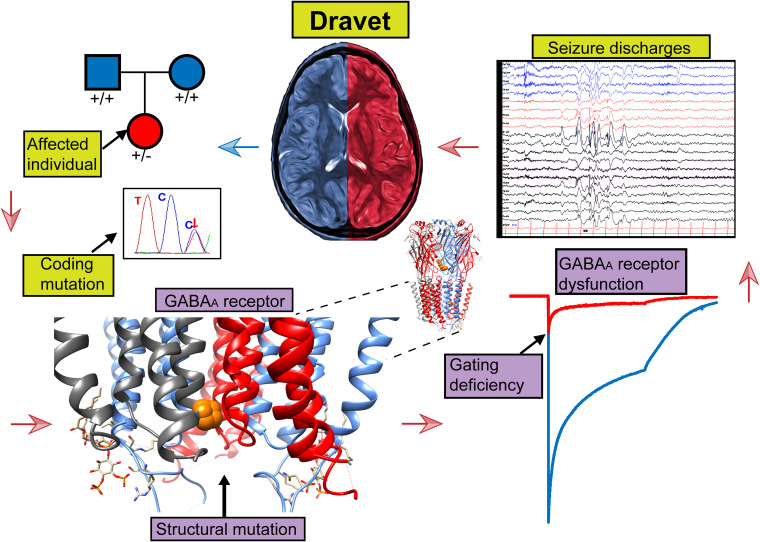

## Introduction

Epileptic encephalopathies (EEs) are a devastating group of severe infantile and childhood onset epilepsies, which are clinically and etiologically heterogeneous and characterized by intractable seizures, neurodevelopmental and cognitive impairment and poor prognosis.^1^ Dravet syndrome is one of the most severe encephalopathies of childhood,^2^^,^^3^ accounting for 1.4% of the cases with severe mental disabilities and early onset epilepsy.^4^^,^^5^ Dravet syndrome is caused mainly by sodium channel gene *SCN1A* variants, but due to the use of massively parallel sequencing technologies, a significant number of variants in other genes such as *GABRs* have been found to arise *de novo* in EE cases including Dravet syndrome patients.^5–9^

GABA_A_ receptors mediate the fastest and most common inhibitory neurotransmission in the central nervous system (CNS). GABA_A_ receptors are heteropentameric ion channels that are assembled from 19 different GABA_A_ receptor subunit subtypes (α1-6, β1-3, γ21-3, δ, ε, ρ1-3, π and θ) and are typically formed with a stoichiometry of 2α, 2β and 1x subunit (where x is a single γ or δ subunit). Binding of GABA to its receptor evokes influx of chloride ions into postsynaptic GABA_A_ receptor channels that usually cause postsynaptic membrane hyperpolarization. The α1β2γ2 receptor is the most abundant GABA_A_ receptor in the CNS.^10^ The α1, β2 and γ2 subunits are abundantly expressed in cortical and thalamic neurons in the corticothalamic circuits that mediate the seizures of genetic epilepsies such as Dravet syndrome. The currently known epilepsy-associated variants identified in GABA_A_ receptor subunits are predominantly distributed in the four genes (*GABRA1*, *GABRB2*, *GABRB3* and *GABRG2*) that code for the most commonly distributed receptor isoforms. A substantial number of missense and nonsense variants in these subunit genes have been associated with autosomal dominant genetic generalized epilepsies (GGEs), ranging from relatively benign childhood absence epilepsy (CAE) to more severe genetic epilepsy with febrile seizures plus (GEFS+), and to EEs including infantile spams (IS), Lenox-Gastaut syndrome (LGS) and Dravet syndrome.^11–14^ *In vitro* cultured cell and *in vivo* animal model studies have shown that variants in these subunits can cause many functional abnormalities, including impaired subunit folding, receptor assembly, receptor trafficking and channel kinetic gating.^15–23^

Using the advantage of next-generation sequencing (NGS) technologies, we discovered nine novel *de novo* variants in *GABRA1*, *GABRB2* and *GABRG2* that were associated with Dravet syndrome and code for subunits that form the most common GABA_A_ receptor (the α1β2γ2 receptor). Four missense variants with *de novo* inheritance were found in *GABRA1* (c.644T>C, p. L215P; c.640C>T, p. R214C; c.859G>A; V287I; c.641G>A, p. R214H), and the patients presented with generalized tonic-clonic seizures (GTCS) and hemiclonic seizures (HS) at an average age at onset of 6 months (Supplementary Table 1). Two missense variants with *de novo* inheritance were found in *GABRG2* (c.269C>G, p. T90R; c.1025C>T, p. P342L), and the patients presented with multiple seizure types including GTCS, HS, myoclonic seizures and episodes of status epilepticus (SE) with onset age of 6 and 8 months, respectively. One in-frame variant with autosomal dominant inheritance (c.990_992del, p.330_331del) and two missense variants with *de novo* inheritance (c.992T>C, p. F331S; c.542A>T, p. Y181F) were found in *GABRB2*, and the patients presented with an average age of onset of 7 months^76^ (Supplementary Table 2). All patients had fever-sensitive seizures and were clinically diagnosed with Dravet syndrome. Interestingly, for all these reported variants, only the *GABRB2* (c.990_992del, p.330_331del) variant did not have *de novo* inheritance; they had autosomal dominant inheritance (from the maternal side). We found that all these variants, regardless which subunit harboured the variant, impaired the function of α1β2γ2 receptors. Our findings agree with the general idea that variants that severely affect the function of any of the subunit subtypes that are part of the α1β2γ2 GABA_A_ receptor are a genetic risk factor for Dravet syndrome epileptogenesis.

## Materials and methods

### Patient phenotypes and Dravet syndrome diagnostic criteria

A cohort with 870 Dravet syndrome patients was recruited from the Department of Pediatrics at the Peking University First Hospital from February 2005 to June 2018. Among 870 Dravet syndrome patients, 743 patients (85.4%) carried *SCN1A* variants, and 9 patients carried *PCDH19* variants. In addition, 14 patients were identified with 6 rare causative genes including 4 with *GABRA1*, 3 with *GABRB2* (reported previously^76^), 2 with *GABRG2*, 1 with *SCN2A*, 2 with *TBC1D24* and 2 with *ALDH7A1* pathogenic variants. To facilitate clarity throughout the manuscript, the nomenclature used for *GABRB2* (c.990_992del, p.330_331del) will be replaced by β2(F331del), designating the change in the protein.

All probands fulfilled the following Dravet syndrome diagnostic criteria: (1) a prolonged unilateral or bilateral clonic or tonic–clonic seizure onset in the first year of life, often triggered by fever (average age of onset was 6 months old); (2) multiple seizure types (myoclonic, focal, atypical absence) in addition to seizures triggered by fever after 1 year of age; (3) usual occurrence of SE; (4) normal early development and subsequent delay in psychomotor development, ataxia and pyramidal signs; (5) normal interictal electroencephalogram (EEG) in the first year of life followed by generalized, focal, or multifocal discharges and (6) seizures refractory to antiepileptic drugs (AEDs). The clinical data collection of this study was approved by the Ethics Committee of Peking University First Hospital. Written informed consent was obtained from the parents of all the patients.

### Epilepsy-gene panel NGS and analysis

Genomic DNA was extracted from peripheral blood lymphocytes of the probands and their parents by a standard method. Samples were screened through an epilepsy-gene panel based NGS. Libraries were first prepared according to the Illumina TruSeq protocols. The captured library was sequenced on an Illumina HiSeq 2500 or X-ten platform for 150 bp pair-end sequencing (Illumina, San Diego, CA). The sequenced reads were mapped to hg19 using the Burrows-Wheeler Aligner (http://bio-bwa.sourceforge.net/bwa.shtml). Reads processing and single-nucleotide variant calling were carried out following the best practice of Genome Analysis Toolkit (GATK version 3.2). Polymorphisms from dbSNP (version 138) and the Exome Aggregation Consortium database were excluded. Variants with putative pathogenicity were further validated by Sanger sequencing. All nine variants identified in the patient were filtered for call quality and frequency in the Genome Aggregation Database (gnomAD). They were all absent from gnomAD, supporting their pathogenicity. We used the American College of Medical Genetics and Genomics (ACMG) guidelines to evaluate the pathogenicity of variants, which indicated they were pathogenic.

### Complementary DNA constructs

The coding sequences of human α1 (*GABRA1*, NM_000806), β2 (*GABRB2*, NM_000813), β3 (*GABRB3*, NM_021912) and γ2L (*GABRG2*, NM_198904.2) GABA_A_ receptor subunits and EGFP were subcloned into pcDNA3.1 expression vectors (Invitrogen). Mutant GABA_A_ receptor subunit constructs were generated using the QuikChange site-directed mutagenesis kit (Agilent Technologies) and confirmed by DNA sequencing.

### Cell culture and transfection of human GABA_A_ receptors

HEK293T cells (ATCC, CRL-11268) were cultured at 37°C in humidified 5% CO_2_ incubator and maintained in Dulbecco's modified Eagle's medium (Invitrogen) supplemented with 10% fetal bovine serum (Life technologies), and 100 IU/ml penicillin/streptomycin (Life Technologies). For expression experiments, 4 × 10^5^ cells were transfected using polyethylenimine (PEI) reagent (40 kD, Polysciences) at a DNA: Transfection reagent ration of 1:2.5 and harvested 36 hours after transfection. To express wt and variant α1β2,3γ2 receptors, a total of 3 µg of α1, β2 or β3 and γ2 subunit cDNAs were transfected at a ratio of 1:1:1 into 6 cm dishes. For the mock-transfected condition, empty pcDNA3.1 vector was added to make a final cDNA transfection amount to 3 μg. For electrophysiology experiments, cells were plated onto 12 mm cover glass slips at 4 × 10^4^ in 35 mm diameter culture dishes, transfected after 24 h with 0.3 μg cDNA of each α1, β2, γ2 L subunits and 0.05 µg of EGFP (to identify transfected cells) using X-tremeGENE HP DNA transfection Reagent (Roche Diagnostics) following manufacturers protocol. Recordings were obtained 48 h after transfection.

### Electrophysiology

Whole-cell recordings of wt and variant GABA_A_ receptor currents were obtained at room temperature from lifted HEK293T cells. The external solution was composed of (in mM): 142 NaCl, 8 KCl, 10 D(+)-glucose, 10 HEPES, 6 MgCl_2_ and 1 CaCl_2_ (pH 7.4, ∼326 mOsm). The internal solution consisted of (in mM): 153 KCl, 10 HEPES, 5 EGTA 2 Mg-ATP and 1 MgCl_2_.6H_2_O (pH 7.3, ∼300 mOsm). GABA (1 mM) was applied for 4 s and 1 ms for measurements of current amplitude and receptor kinetic properties. The currents were recorded using an Axopatch 200B amplifier (Axon Instruments), low-pass filtered at 2 kHz using the internal 4-Pole Bessel filter of the amplifier, digitized at 10 kHz with Digidata 1550 (Axon Instruments) and stored for offline analysis as previously described.^24^

### Western blot and surface biotinylation

HEK293T cells were collected in modified Radioimmunoprecipitation assay (RIPA) buffer [50 mM Tris (pH = 7.4), 150 mM NaCl, 1% NP-40, 0.2% sodium deoxycholate, 1 mM EDTA] and 1% protease inhibitor cocktail (Sigma). Collected samples were subjected to gel electrophoresis using 4–12% BisTris NuPAGE precast gels (Invitrogen) and transferred to Polyvinylidene difluoride fluorescence-based (PVDF-FL) membranes (Millipore). Primary antibodies used to detect GABA_A_ receptors were as the follows: Mouse α1 subunit antibody (1:500; NeuroMab, 75–136), rabbit β2 subunit antibody (1:1000; Millipore, AB5561), rabbit β3 subunit antibody (1:500; Novus, NB300-199), and rabbit γ2 subunit antibody (1:500; Millipore, AB5559). The Mouse anti-Na^+^/K^+^ ATPase antibody (1:000; DSHB, a6F) was used as a loading control. IRDye^®^ (LI-COR Biosciences) conjugated secondary antibody was used at a 1:10 000 dilution in all cases. Membranes were scanned using the Odyssey Infrared Imaging System (LI-COR Biosciences). The integrated intensity value of bands was determined using the Odyssey Image Studio software (LI-COR Biosciences).

Biotinylation protocols have been described previously.^21^ Briefly, transfected cells were incubated in membrane-impermeable reagent sulf-HNS-SS-biotin (1 mg/ml, Thermo Scientific) at 4°C for 40 min. Cells were lysed after being quenched with 0.1 M glycine. Lysates were cleared by after centrifugation and then incubated overnight with High Binding Capacity NeutrAvidin beads (Thermo Scientific Pierce). After incubation, protein was eluted in sampling buffer (Invitrogen) containing 10% β-mercaptoethanol and subjected to immunoblotting.

### Confocal microscopy

For immunofluorescence, cover slip grown HEK293T cells were washed with phosphate-buffered saline (PBS) and fixed with Prefer (Anatech) for 20 min. To stain total proteins, cells were treated with 0.5% Triton X-100 for 5 min. The fixed/permeabilized cells were blocked for 2 h with 5% bovine serum albumin in PBS, and then stained with primary antibodies either overnight at 4° or for 2 h at room temperature, followed by incubation in Alexa Fluor 488-conjugated donkey anti-mouse IgG antibodies and Alexa Fluor 568-conjugated donkey anti-rabbit IgG antibodies for another 2 h at room temperature. Primary antibodies used were as the follows: rabbit anti-HA (Cell Signaling, C29F4), mouse purified anti-HA (BioLegend, 16B12), mouse monoclonal anti-α1 subunit (Millipore, MAB339), rabbit polyclonal anti-α1 subunit (Millipore, 06–868), mouse monoclonal anti-β2/3 subunit (Millipore, 62-3G1), mouse monoclonal anti-calnexin (Abcam, ab22595). Coverslips were mounted with Prolong Gold antifade reagent (Thermo Fisher Scientific Inc.).

Confocal images were obtained from immunostained cells using a Zeiss LSM 510 Meta inverted confocal microscope. Stained HEK293T cells were excited with the 488 nm laser for the Alexa 488 fluorophore signal and the 543 nm laser for the 568-fluorophore signal. Images were taken with 12 bit, 1024 × 1024 pixel resolution. Pinholes were adjusted so that the sample thickness was 0.9 μm. An average of four scans was taken to decrease the background noise. Confocal experiments were performed in part using the Vanderbilt University Medical Center Cell Imaging Shared Resource.

Colocalization analysis was performed using the Coloc2 plugin in the open source image processing program Fiji.^25^ Microscopic image files were imported, and the two channels (green and red) were separated. The two channels being compared were assigned to channel 1 (green) and channel 2 (red) in a manner consistent across all samples. A region of interest surrounding individual cells was selected in the green channel, and its location was set in the Coloc2 panel. Both Pearson’s correlation coefficient (R) and Manders’ colocalization coefficient (MCC) were calculated.

### Docking of PIP_2_

The α1 and β3 subunits of the cryo-EM structure of the human pentameric α1β3γ2L GABA_A_ receptor (PDB 6HUO^26^) were used as starting models for our simulations. A deletion at the homologous position of F331 of the β2 subunit was inserted in the β3 subunit of the structure, and the mutant β3 subunit was labelled as the β3(F332del) subunit. Wt and mutant β3(F332del)α1 subunit dimers were input into ROSIE (rosie.graylab.jhu.edu) using the RossettaBackrub flexible backbone to identified structural models (backbones) with side chain residues with a tolerated profile at the β3/α1 interface.^27^ The highest-ranked solutions from 1 to 10 independent simulation runs with a root mean square deviation (RMSD) below 2.0 Å were selected for molecular docking. RMSD of the top 10 mutated structures when compared with the RMSD to the cryo-EM native structure (wt) was 0.94 ± 0.04 Å. PatchDock, a molecular docking method based on shape complementary functions,^28^ was used to identified ligand-binding modes of phosphatidylinositol-4,5-bisphosphate (PIP_2_) at the interfaces of the wt and mutant β3α1 dimers. Docking accuracy of 20–40 independent complexes was analysed by Molegro Virtual Docker (MVD).^29^ Complexes were defined as bound PIP_2_ to wt and mutant receptors. Afterwards, MVD optimized the orientation of any rotatable hydrogens on the ligand and protein, which were involved in hydrogen bonds within the complexes. To further increase docking accuracy, the complexes were reranked by performing an energy minimization of the current ligand and taking into account the total atom energy of the complex, which was the summation of the pairwise steric and hydrogen bonding energy, the pairwise electrostatic interactions, and the internal ligand energy. We prepared the figures using Chimera 1.7.^30^

### Statistical analysis

Numerical data were reported as mean ± SEM. For electrophysiological experiments, data points represent the mean ± SEM from 5 to 23 different patched cells per experimental condition acquired in two different experimental sessions (Table 1). Statistical analyses were performed using GraphPad Prism (GraphPad Software 8.2). Statistically significant differences were taken as *P* < 0.05 using one-way ANOVA followed by Dunnett’s multiple comparison test and unpaired two-tailed Student's *t* test when appropriate.

**Table 1:** Effects of Dravet syndrome-associated variants on α1β2γ2L receptor function

	α1β2γ2L	α1R214C	α1L215P	α1V287I	β2Y181F	β2F331S	β2F331del	γ2T90R
Current amplitude, pA	4769 ± 160	1583 ± 201^a^	1547 ± 161^a^	1613 ± 49^a^	4872 ± 159^b^	4448 ± 65^c^	4048 ± 82^d^	652 ± 17^a^
	(*n* = 23)	(*n* = 8)	(*n* = 12)	(*n* = 6)	(*n* = 7)	(*n* = 17)	(*n* = 10)	(*n* = 10)
Desensitization extent, %	68 ± 2	85 ± 2^a^	57 ± 2^e^	50 ± 4^a^	54 ± 2^a^	73 ± 1^f^	69 ± 1^g^	82 ± 1^a^
	(*n* = 13)	(*n* = 8)	(*n* = 12)	(*n* = 6)	(*n* = 11)	(*n* = 10)	(*n* = 10)	(*n* = 10)
Desensitization τ, ms	815 ± 47	602 ± 37^h^	1763 ± 161^a^	1725 ± 140^a^	2378 ± 157^a^	1114 ± 32^i^	1336 ± 33^j^	353 ± 55^k^
	(*n* = 13)	(*n* = 8)	(*n* = 12)	(*n* = 5)	(*n* = 11)	(*n* = 10)	(*n* = 10)	(*n* = 10)
Activation τ, ms	076 ± 0.05	1.39 ± 0.08^l^	1.65 ± 0.09^m^	0.46 ± 0.02^n^	2.84 ± 0.27^a^	0.91 ± 0.04^o^	0.80 ± 0.08^p^	3.41 ± 0.38^a^
	(*n* = 13)	(*n* = 8)	(*n* = 12)	(*n* = 7)	(*n* = 11)	(*n* = 10)	(*n* = 10)	(*n* = 10)
Deactivation τ, ms	1160 ± 67	451 ± 31^a^	329 ± 17^a^	1375 ± 124^q^	400 ± 18^a^	1038 ± 37^r^	2248 ± 123^a^	1081 ± 138^s^
	(*n* = 13)	(*n* = 8)	(*n* = 11)	(*n* = 6)	(*n* = 11)	(*n* = 10)	(*n* = 10)	(*n* = 10)

Macroscopic parameters were obtained from lifted cells voltage-clamped at −20 mV when applying 1 mM GABA for 4 s. Data points represent the mean ± S.E.M from 5 to 23 different patched cells per experimental condition acquired in two different experimental sessions. One-way ANOVA with Dunnett’s multiple comparisons test was used to determine significance relative to α1β2γ2L.

a *P* < 0.0001.

b *P* = 0.997.

c *P* = 0.262.

d *P* = 0.0019.

e *P* = 0.0003.

f *P* = 0.150.

g *P* = 0.994.

h *P* = 0.573.

i *P* = 0.163.

j *P* = 0.0019.

k *P* = 0.0073.

l *P* = 0.085.

m *P* = 0.0015.

n *P* = 0.817.

o *P* = 0.989.

p *P* = 0.999.

q *P* = 0.384.

r *P* = 0.789.

s *P* = 0.968.

### Data availability

The data that support the findings of this study are available from the corresponding author upon request by qualified researchers for non-commercial research purposes.

## Results

### *De novo* and maternal familial autosomal dominant *GABR* variants were identified in nine individuals with Dravet syndrome

**Figure 1:**
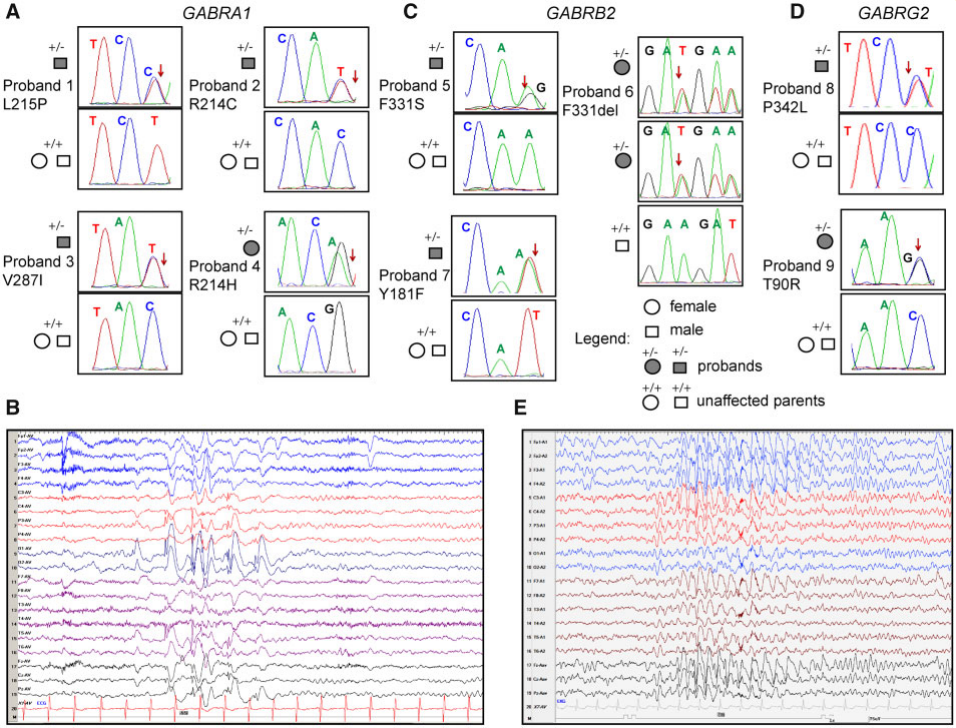
**Dravet syndrome patient phenotypes.** Pedigree and segregation analysis in nine Dravet syndrome patients of the nine *GABR* missense variants identified in (**A)** *GABRA1*, (**C)** *GABRB2* and (**D)** *GABRG2*. Arrows indicate the position of the variant in the Sanger chromatograms of the affected probands. (**B**) Representative EEGs recorded during a seizure at 7 years of age of proband 1 showing high amplitude spike-wave discharges in the left occipital leads. (**E**) Representative EEG recorded during a seizure at 12 years of age of proband 9 showing generalized spike waves.

The clinical features and family pedigrees of the nine probands with Dravet syndrome and *GABR* variants were summarized (Supplementary Tables 1 and 2 and Fig 1A, C, D). Of the nine Dravet syndrome patients, eight had *de novo* inheritance and one had autosomal dominant maternal inheritance. In general, the seizure at onset was either a GTCS or a HS that lasted from 1 to 25 min, mainly with fever, and which were caused either after vaccination or a hot shower. The seizures of the nine patients were fever-sensitive and one patient was light-sensitive. In addition, all nine patients also had episodes of SE. Multiple types of seizures appeared after 1 year of age. Overall, all nine patients had HS, eight had GTCSs, seven had myoclonic seizures, five had focal seizures and three had atypical absence seizures. EEGs of the nine patients were normal at an early ages, then generalized, focal or multifocal spike wave discharges were detected (Fig 1B and E). In addition, brain magnetic resonance imagings were normal in all probands, except for proband 9, which presented bilateral ventricular enlargement. The seizures of the nine patients were mostly drug-resistant, with all patients receiving two or more AEDs. Among the AED of choice, oxcarbazepine exacerbated the seizures of probands 2, 3, 4, 7 and 8. Proband 2 had been seizure-free for 6 years, but relapsed after withdrawal of AED, while proband 6 had been seizure-free for 6 years and 6 months. However, the early clinical characteristics of probands 2 and 6 coincided with the diagnosis of Dravet syndrome. All nine patients had mild to moderate mental deficiency at the last follow-up.

### *GABR* variants mapped to the N-terminal and pore domains of the GABA_A_ receptor are likely pathogenic.

**Figure 2:**
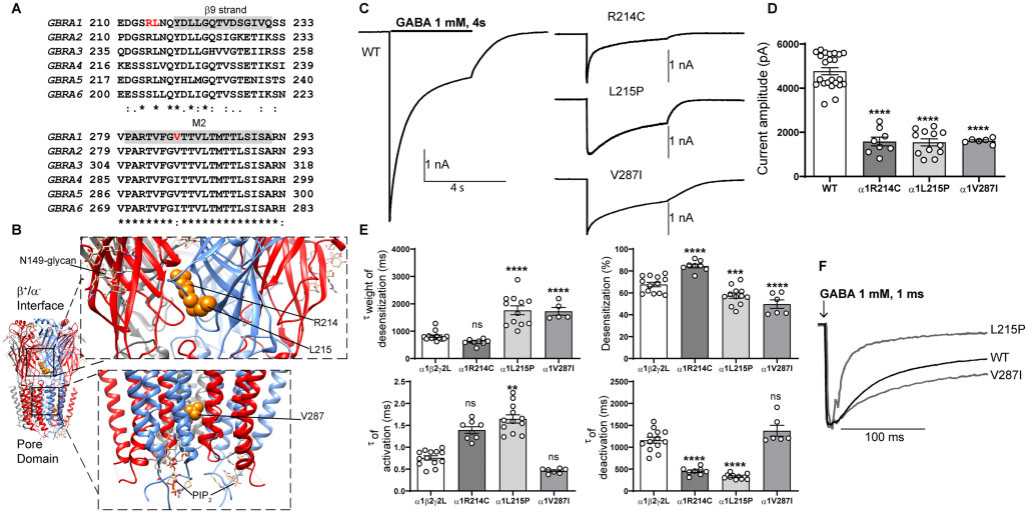
**Electrophysiology and structural mapping of Dravet syndrome *GABRA1* variants.** (**A**) Alignment of human α1-6 GABA_A_ receptor subunits. Positions of *de novo* variants in the α1 subunit are shown in red. Across all sequences, the residue L215 is identical (*), and the residue V287 is conserved (:). The β9-strand (highlighted in *grey*) and the transmembrane domain M2 (highlighted in *grey*) are represented across subunits above the alignments. (**B**) The cryo-EM structure of the human pentameric α1β3γ2L GABA_A_ receptor was used for N-terminal and pore domain views (PDB 6HUO).^26^ Subunits α1 (*blue*), β3 (*red*) and γ2L (*grey*) are shown. The principal (+) and complementary (−) faces of α1, β3 and γ2 subunits and binding sites for N-linked glycans and phosphatidylinositol-4,5-bisphosphate (PIP_2_) are indicated. *GABRA1 de novo* variants are mapped onto the α1 subunit and represented in orange. (**C**) Representative GABA-evoked-current traces were obtained following rapid application of 1 mM GABA for 4s to lifted HEK293T cells expressing wt α1 or variant α1(R214C, L215P, V287I) subunit-containing α1β2γ2L GABA_A_ receptors. (**D**) Peak amplitudes of wt α1 or variant α1(R214C, L215P, V287I)β3γ2L subunit-containing α1β2γ2L GABA_A_ receptors. (**E**) Bar graphs are presented showing desensitization, activation and deactivation of α1β2γ2L GABA_A_ receptors containing wt α1 or variant α1(R214C, L215P, V287I)β3γ2L subunits. (**F**) Representative normalized GABA-evoked current traces illustrate the differences in the deactivation rates of α1β2γ2L GABA_A_ receptors containing wt α1 or variant α1(L215P, V287I) subunits currents after rapid application of 1 mM GABA for 1 ms. Data points represent the mean ± SEM from 5 to 23 different patched cells per experimental condition acquired in two different experimental sessions (Table 1). One-way ANOVA with Dunnett’s multiple comparisons test was used to determine significance relative to α1β2γ2L (WT). ****P < 0.0001, ***P < 0.001, **P < 0.01, and ^ns^*P* > 0.05, respectively.

The crystal structure of the human α1β3γ2 receptor revealed that all five subunits contribute to a large extracellular N-terminal domain that contains the binding sites for GABA, diazepam, PIP_2_ and other allosteric compounds and four transmembrane helices (M1-M4) (Figs 2B and 4B). The M2 helices from the pore domain that surrounds the conduction pathway.^31^ By comparing the amino acid sequence alignments of the GABA_A_ receptor α, β and γ subunits (Figs 2A, 4A and 5A), the Dravet syndrome-associated variants reported in this study were mapped mainly within the N-terminal domain or the transmembrane M2 pore and M3 domains (Figs 2B and 4B). This is a significant finding considering the importance of these domains in the function of the receptor.^32^

### *GABRA1* Dravet syndrome variants

The α1 subunit missense variants, R214C, R214H and L215P, occurred upstream of the β9-strand, lining up with residues at the β/α interfaces, and the α1 subunit variant V287I was mapped to the 5ʹ position of the M2 helix that faces the conduction pore of the receptor (Fig. 2A and B). The R214 and V287 residues were both conserved in four of the six α subtypes (α1, α2, α3 and α6), while the L215 residue was conserved in all six α subtypes (Fig. 2). Introduction of each of the variant residues was predicted to be deleterious in *in silico* analysis using Polyphen-2^33^ and SIFT^34^ programs.

The R214H variant was previously reported in two unrelated cases of early infantile EE and Dravet syndrome,^7^ and functional studies in oocytes classified it as a loss-of-function variant. On the other hand, although the R214C variant has not been reported in individuals suffering from an EE, this variant was found in a patient referred to GeneDx (www.genedx.com) for epilepsy tests, and therefore, was entered into the ClinVar database without clinical information (Variation ID: 265161. NM_000806.5: C.640C>T). The L215P and V287I variants have not been reported as EE-causing variants. However, the L215V variant was found in a sporadic case with unclassified seizures.^35^ Moreover, the ClinVar database contains two entries for variants at the V287 position, leucine (Variation ID: 430503) and isoleucine (Variation ID: 205522) (Supplementary Table 1). The V287L (NM_000806.5: C.859G>T) variant was reported in a case with early onset epileptic encephalopathy (EOEE),^9^ while the V287I (NM_000806.5: C.859G>A) variant was found in a childhood-onset epilepsy panel (GeneDx).

#### The *de novo* variant α1 subunits decreased GABA-evoked currents from α1β2γ2 receptors

We determined the functional consequences of Dravet syndrome-associated variant α1 subunits by measuring macroscopic GABA-evoked currents from lifted HEK293T cells coexpressing wt or variant α1 subunits with β2 and γ2 subunits. We measured the peak current amplitudes from receptors expressed on the cell surface by applying 1 mM GABA for 4 s (Fig. 2C). The α1β2γ2 receptors containing the variant α1(R214C), α1(L215P) or α1(V287I) subunit decreased GABA-evoked currents by ∼60% (*P *<* *0.0001, Table 1) (Fig 2C and D).

We further examined whether the α1 subunit Dravet syndrome variants impaired channel gating by recording macroscopic kinetic properties of GABA-evoked currents (Fig. 2C). We measured current desensitization rates and extents, activation rates and deactivation rates of wt α1β2γ2 currents and currents from α1β2γ2 receptors containing variant α1 subunits (Fig. 2E). GABA_A_ receptor current desensitization during a 4 s GABA (1 mM) application was slowed by the variant α1(L215P) and α1(V287I) subunits but was unchanged by the variant α1(R214C) subunit (Fig. 2E). The α1(L215P) and α1(V287I) subunit variants decreased (*P *=* *0.003 and *P *<* *0.0001, Table 1) (Fig. 2E) and the α1(R214C) variant increased (*P *<* *0.0001, Table 1) the extent of current desensitization (Fig. 2E). In addition, the activation and deactivation rates were inversely correlated. Receptors with variant α1(R214C) and α1(L215P) subunits differently affected activation rates (*P *=* *0.085 and *P *=* *0.0015, Table 1) and accelerated deactivation (*P *<* *0.0001, Table 1), while the variant α1(V287I) subunit did not change activation (*P *=* *0.817, Table 1) or deactivation (*P *=* *0.384, Table 1).

Figure 2F showed the differences in variant α1(L215P) and α1(V287I) subunits on current deactivation measured at current offset from a 1 ms GABA (1 mM) application. These results demonstrate that the *GABRA1* R214C and L215P variants at the β/α interface of the GABA-binding domain affected both activation and desensitization rates of the receptor. On the other hand, the *GABRA1* V287I variant that is mapped after the activation gate of the receptor, exclusively affected the receptor desensitization. These findings strongly confirmed the close relationship of receptor function and the location of variants in conserved structural domains of the GABA_A_ receptor.^32^^,^^36^

#### The *de novo* variant α1 subunits did not alter GABA_A_ receptor surface or total cell expression

**Figure 3:**
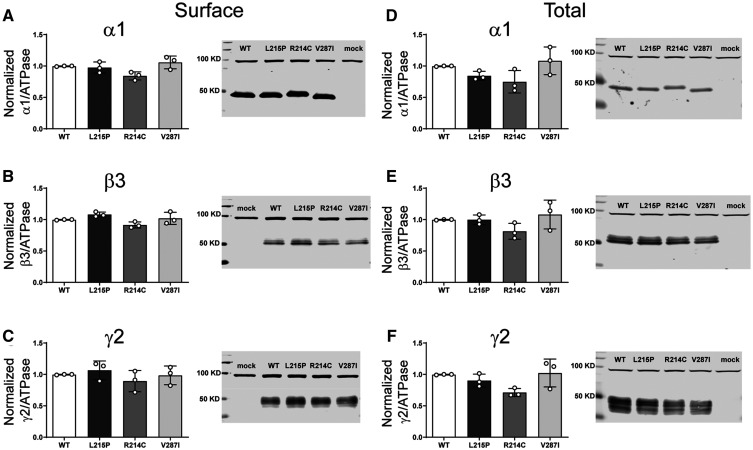
**Surface and total expression of Dravet syndrome *GABRA1* variants.** Wt α1 or variant α1(L215P, R214C and V287I) subunits were coexpressed with β3 and γ2 subunits in HEK293T cells. Surface receptors were biotinylated and stained against anti-GABA_A_ receptor (**A**) α1, (**B**) β3 and (**C**) γ2 subunits. Control loading was assayed using anti-ATPase antibodies. Total cell lysates were collected, analysed by SDS-PAGE and blotted by anti- (**D**) α1, (**E**) β3, (**F**) γ2 subunit and anti-ATPase antibodies for loading controls. Representative western blots were presented at the right of the panels. Band intensities of the α1, β3 and γ2 subunits were normalized to the ATPase signal. Mock refers to the transfection with an empty plasmid. Values reported are mean ± SEM (Supplementary Table 3). One-way ANOVA followed by Dunnett’s multiple comparison test was used to determine significance relative to α1β2γ2L (WT). No significance was shown (*p* > 0.05). Corresponding uncropped blots are available in the Supplementary data.

Decreased current amplitudes can be produced by defective receptor channel gating and/or pore conductance or by impaired receptor biogenesis. Thus, we assessed surface trafficking of variant α1 subunit-containing α1β3γ2 receptors by cotransfecting HEK293T cells with β3, γ2 and wt or variant α1 subunits at a 1:1:1 α1:β3:γ2 subunit ratio and evaluated surface levels of wt and variant α1 subunits and of wt β3, and γ2 subunits by surface biotinylation (Fig. 3A–C). Compared to coexpressed wt α1 subunits, we found no differences in surface levels of variant α1 or of wt β3 or γ2 partnering subunits, which confirmed that they were assembled and expressed as pentameric αβγ receptors on the cell surface and that no dominant negative effects on wt or variant subunits were observed (Supplementary Table 3). In addition, none of the α1 subunit variants changed total levels of α1, β3 or γ2 subunits in whole cell lysates (Fig. 3D–F), supporting the lack of effect of these α1 variants on biogenesis of the receptor.

### *GABRB2* Dravet syndrome variants

**Figure 4:**
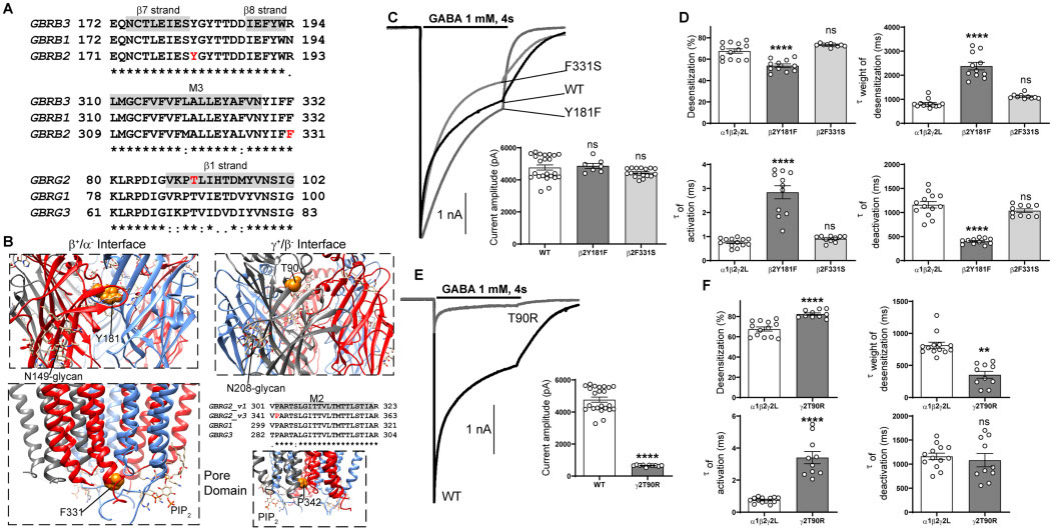
**Electrophysiology and structural mapping of Dravet syndrome *GABRB2* and *GABRG2* variants.** (**A**) Alignment of human β1-3 and γ1-3 GABA_A_ receptor subunits and positions of *de novo* variants in the β2 and γ2 subunits are shown in *red*. The β2(Y181, F331) and γ2(T90) residues are identical (*) across all subunit sequences. The β7, β8 and β1-strands (highlighted in *grey*) and the transmembrane domain M3 (highlighted in *grey*) are represented above the alignments. (**B**) In the *top* panels, *GABRB2* and *GABRG2 de novo* variants (in *orange*) are mapped onto the β (in *red*) and γ (in *grey*) subunits at the β/α and γ/β interfaces of the cryo-EM structure of the human pentameric α1β3γ2L GABA_A_ receptor (PDB 6HUO).^26^ Proximity to binding sites for N-linked glycans and PIP_2_ are indicated. In the *bottom left* panel, the *GABRB2 de novo* variant F331 (in *orange*) is mapped onto the β (in *red*) subunit of the receptor. The *bottom right* panels show the alignment of the pore-lining residues of M2 of the γ subunits and the pore domain where the γ2__v3_P342L subunit is located in the M2 domain (highlighted in *grey*) of the receptor. (**C**) Representative non-normalized currents from α1β2γ2L receptors containing wt β2 or variant β2(Y181F, F331S) subunits. Inset bar graphs to the right show the average peak current recorded from those cells. (**D**) B*ar* graphs comparing desensitization, activation and deactivation of α1β2γ2L receptor currents from GABA_A_ receptors containing wt β2 or variant β2(Y181F, F331S) subunits. (**E**) Representative non-normalized α1β2γ2L receptor currents containing wt γ2 or variant γ2(T90R) subunits *Inset* bar graphs to the right show the average peak currents recorded from those cells. (**F**) B*ar* graphs comparing desensitization, activation and deactivation of α1β2γ2L receptor currents from GABA_A_ receptors containing wt γ2 or variant γ2(T90R) subunits. Data points represent the mean ± SEM from 7 to 23 different patched cells per experimental condition acquired in two different experimental sessions (Table 1). One-way ANOVA with Dunnett’s multiple comparisons test and unpaired two-tailed Student's *t* test were used to determine significance relative to α1β2γ2L (WT). ****p < 0.0001, **p < 0.01, and ^ns^*p* > 0.05, respectively.

The β2 subunit variant Y181F was mapped to the N-terminal domain in the β7-β8 loop and β1-strand (Fig. 4A and B), whereas the β2 subunit variant F331S was in the M3 helix of the receptor (Fig. 4A and B). These residues are conserved across all GABA_A_ receptor β subunits (Fig. 4A), and the variants are also predicted to be deleterious in *in silico* analyses. Neither of these variants have been reported to be associated with cases of EE (Supplementary Table 2).

#### The *de novo* variant β2(Y181F) subunit, but not the β2(F331S) subunit, mainly altered macroscopic kinetic properties

Unlike the Dravet syndrome-associated variants in the α1 subunit, the β2 subunit missense variants Y181F and F331S did not affect peak GABA-evoked current amplitudes when compared to wt receptor peak currents (*P *=* *0.997 and *P *=* *0.262, Table 1) (Fig. 4C). We measured the desensitization rates and extents, activation rates and deactivation rates of the GABA_A_ receptor currents by coexpressing wt α1 and γ2 subunits with wt β2 or variant β2(Y181F) or β2(F331S) subunits (Fig. 4D).

We found that currents from β2(Y181F) subunit-containing receptors had significantly decreased desensitization extent (*P *<* *0.0001, Table 1) and slowed desensitization (*P *<* *0.0001, Table 1). Currents from receptors containing the variant β2(Y181F) subunit also had slowed activation and faster deactivation rates (*P *<* *0.0001, Table 1).

In contrast, currents from receptors containing the variant β2(F331S) subunit had unchanged desensitization time course or desensitization extent of the current (*P *=* *0.163 and *P *=* *0.150, Table 1), and the variant β2(F331S) subunit produced no change in activation or deactivation rates (*P *=* *0.989 and *P *=* *0.789, Table 1).

Thus, similar to the results observed with the *GABRA1* variants at the β/α interface of the GABA-binding domain, the variant β2(Y181F) subunit altered both activation and desensitization rates of the receptor. In contrast, the variant β2(F331S) subunit at the edge of M3 had no apparent effects on the kinetic properties of the receptor.

#### The variant β2(F331del) subunit had maternal familial inheritance

**Figure 5:**
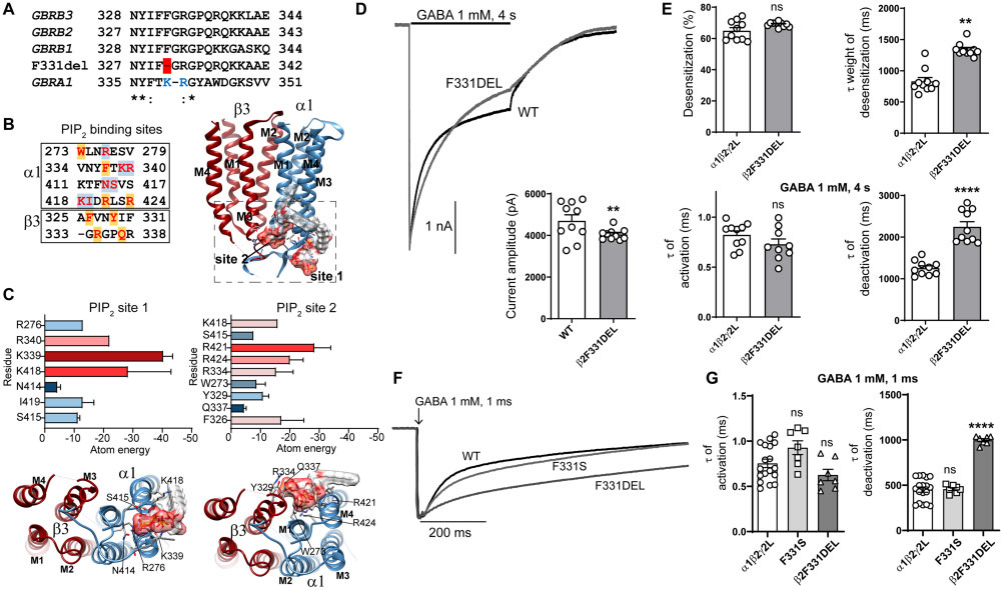
**Electrophysiology and structural mapping of Dravet syndrome *GABRB2* F331del.** (**A**) Alignments of the edge of M3 of the human β1-3subunits, the β2(F331del) and α1 subunits. The position of the deletion is shown in *red*, and the PIP_2_ binding site residues in *blue* as reported.^26^ (**B**) *Left*, amino acids predicted to be part of the network of interactions of PIP_2_ in both α1 and β3(F332del) subunits are displayed in *red*. Highlighted in *blue* is shown the predicted residues of being part of the binding site 1 of PIP_2_, and in *orange*, the residues predicted for the binding site 2 of PIP_2_. *Right*, TM domains of the β3(F332del)α1 dimer enclosed the two docking PIP_2_ binding sites. PIP_2_ is in molecular surface electrostatic representation. (**C**) *Top left*, relevant residues that contributed to the network of interactions at the binding site 1 of PIP_2_ are classified according to total atomic energy (Kcal/mol), the weakest binding being blue, and the strongest red. *Bottom left*, intracellular view of the binding site 1 of PIP_2_. *Top right*, relevant residues that contributed to the network of interactions at the binding site 2 of PIP_2_ are classified as indicated before. *Bottom right*, intracellular view of the binding site 2 of PIP_2_. (**D**) Representative GABA-evoked-current traces evoked by 1 mM GABA for 4 s to cells express α1β2γ2L receptors with wt β2 or variant β2(F331del) subunits. *Bottom right* show the average peak current recorded from those cells. (**E**) Bar graphs displaying the effects of wt and variant subunits on macroscopic kinetics of GABA_A_ receptors evoked by 1 mM GABA for 4 s. (**F**) Representative normalized GABA-evoked currents from α1β2γ2L receptors containing wt β2 or variant β2(F331del, F331S) subunits illustrate the differences in the deactivation rates of wt and variant β2(F331del) and β2(F331S) receptor currents after rapid application of 1 mM GABA for 1 ms. (**G**) Comparison of the effects of wt and variant β2(F331del, F331S) subunits on macroscopic kinetics of GABA_A_ receptors after rapid application of 1 mM GABA for 1 ms. Data points represent the mean ± SEM from 6 to 18 different patched cells per experimental condition acquired in two different experimental sessions (Table 1). One-way ANOVA with Dunnett’s multiple comparisons test and unpaired two-tailed Student's *t* test were used to determine significance relative to α1β2γ2L (WT). ****P < 0.0001, **P < 0.01, and ^ns^*P* > 0.05, respectively.

In contrast to the other eight Dravet syndrome variants that had *de novo* inheritance, the β2(F331del) subunit variant, a deletion of a single nucleotide that resulted in an in-frame deletion, was familial with maternal inheritance (Fig. 1C). The residue phenylalanine (Phe; F) coded by TTT was deleted in the 331 position, and the β2 subunit protein product was missing one amino acid, a Phe. Based on the cryo-EM *GABAR* structure (Fig. 4B),^26^^,^^31^ the β2(F331) subunit residue is structurally located at the cytoplasmic interface of M3 in the β subunit, which is the homologous site of PIP_2_ binding to the α1 subunit (Fig. 5A and B). In contrast to the β2 subunit variant F331S, the β2 subunit variant F331del is an in-frame deletion that predicts shortening of the edge of M3 by one residue. Throughout the alignment of the α1 and β subunits and the deletion, the β2-R333 (β3-R334) subunit aligns with the binding site of PIP_2_ in the α1(K339, R340) subunit (Fig. 5A). To gain insight into whether this shortening of the β subunit favours a network of PIP_2_ interactions with the arginine that was revealed on the interface of M3, structural docking models of PIP_2_ and the mutant GABA_A_ receptor were simulated. Our simulations found that, in contrast to the wt receptor, PIP_2_ will bind at two sites in the mutant receptor. A binding site which corresponds to the PIP_2_ binding site in the α1 subunit (PIP_2_ site 1), and an accessory site at the homologous interface of the β3 subunit where the deletion occurs (PIP_2_ site 2) (Fig. 5B and C). Comparisons of the residues that are part of the network of interactions of the PIP_2_ binding site in the cryo-EM structure (6HUO) confirmed that the predicted residues at site 1 were almost identical, with strong interactions towards three charged residues in the α subunit (R340, K339, K418) (Fig. 5B and C). Consistently, the PIP_2_ site 1 was mapped on a surface cavity between M3 and M4 helices of the α1 subunit as reported.^26^^,^^31^ The mutated receptor also predicted a secondary site but at the β/α interface where a network of interactions between additional charged residues between the β3 (R334) and α1 (K418, R421, R424) subunits were favoured (Fig. 5B and C). The PIP_2_ site 2 mapped onto the β M3 helix and α M1 and α M4 helices. Since PIP_2_ regulates the function of various channels and receptors,^37^ these findings suggested a mechanistic basis for the effect of mutations/variants in these regions on differential effects on the macroscopic kinetics of the receptor, and it could be correlated with deficits in PIP_2_ binding.

To further determine whether the structural differences predicted by the β2 subunit variant F331del decreased receptor function, the peak current amplitudes and macroscopic kinetics of β2(F331del) subunit-containing receptors coexpressed on the cell surface were measured by applying 1 mM GABA for 4 s (Fig. 5D and E). In contrast with the missense variant β2(F331S) that displayed no defects (Fig. 4C and D), the β2(F331del) subunit decreased peak GABA-evoked currents (*P *=* *0.0019, Table 1), increased desensitization rates (*P *=* *0.0019, Table 1) and deactivation rates (*P *<* *0.0001, Table 1) but produced no changes in desensitization extent of the current or rate of current activation (*P *=* *0.994 and *P *=* *0.999, Table 1).

Further, we compared the differences of β2 subunit variants F331S and F331del on current activation and deactivation measured at current offset of a 1 ms GABA (1 mM) application (Fig. 5F). Unexpectedly, the β2 subunit variant F331del (998 ± 17 ms, *n* = 7, *P *<* *0.0001) slowed deactivation of the receptor up to two times the difference of the wt receptor or the variant β2(F331S) subunit-containing receptor (451 ± 29 ms, *n* = 18; 457 ± 17 ms, *n* = 7), with no differences in activation of the receptor (wt 0.75 ± 0.05 ms, *n* = 18; F331S 0.93 ± 0.08 ms, *P *=* *0.096, *n* = 7; F331del 0.63 ± 0.06 ms, *n* = 7, *P *=* *0.256) (Fig. 5G).

#### β2 subunit variants minimally altered GABA_A_ receptor surface, but not total cell surface expression

**Figure 6:**
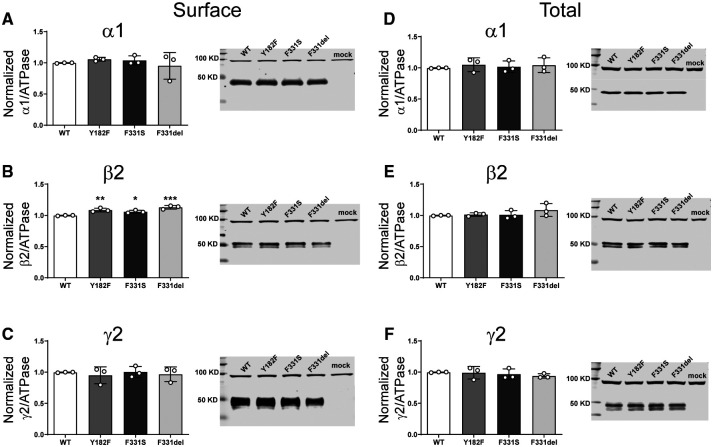
**Surface and total expression of Dravet Syndrome *GABRB2* variants.** Wt β2 or variant β2(F331del) subunits were coexpressed with α1 and γ2 subunits in HEK293T cells. Surface (**A**, **B**, **C**) and total expression (**D**, **E**, **F**) were assessed as shown in Fig. 3. Values reported are mean ± SEM (Supplementary Table 3). One-way ANOVA followed by Dunnett’s multiple comparison test was used to determine significance relative to wild type (WT). ***P < 0.001, **P < 0.01 and *P < 0.05, respectively. Corresponding uncropped blots are available in the Supplementary data.

To determine whether the β2(Y181F), β2(F331S) and β2(F331del) variant subunits affected the biogenesis and/or trafficking of variant GABA_A_ receptors, we measured surface and total expression of wt β2 and variant β2 subunit-containing α1β2γ2 receptors (Fig. 6). None of the variant subunits reduced surface (Fig. 6A–C) or total (Fig. 6D–F) levels of α1, β2 or γ2 subunits (Supplementary Table 3). Unexpectedly, the β2(Y181F), β2(F331S) and β2(F331del) variant subunits significantly increased surface β2 subunit levels slightly (Fig. 6B), without altering α1 or γ2 subunit levels (Figs 6A and C). These results are puzzling since it is well known that the β2 subunit does not traffic alone to the membrane.^38^ It is not clear what would be the result of an increase of these subunits on the surface. Whether they are favouring the formation of binary αβ receptors or pentameric receptors with different stoichiometries than the wt receptors, the contribution to the whole currents seemed to be minimal, only a dysfunction of the macroscopic kinetics.

### *GABRG2* Dravet syndrome variants

The γ2 subunit variant T90R was mapped to the N-terminal domain in the β7–β8 loop and β1-strand (Fig. 4A and B). The residue was highly conserved across all GABA_A_ receptor subunits, and the variant was predicted to be deleterious in *in silico* analyses. This variant has not been reported to be associated with cases of EE (Supplementary Table 2). The γ2 subunit P342L variant found in transcript variant 3 (NM_198903.2), corresponds to the P302L variant reported in the transcript variants 1 and 2 associated with Dravet syndrome.^39^ In addition, a substitution of a methionine at the 90 position of the γ2(T90M) subunit (NM_198903.2: C.269C>T) variant was reported as a *de novo* variant in an affected patient with GEFS+ and CAE.^40^ The corresponding variant was reported in the ClinVar database and classified as likely pathogenic (Variation ID: 379114).

#### The *de novo* variant γ2(T90R) subunit decreased GABA-evoked currents and had robust dominant negative effects

The γ2(T90R) subunit variant introduced a positively charged residue at the only γ+/β- interface of the receptor, thus imposing a large polar side chain within the α1-β2 loop in the extracellular domain of the receptor (Fig. 4B, top right). At this location, homologous assembly motifs within the subunits contribute to proper oligomerization among the γ+/β-, β+/α- and α+/γ- interfaces of pentameric receptors and receptor trafficking to the cell surface.^41–44^

To evaluate whether the variant γ2(T90R) subunit could assemble with α and β subunits and traffic to cell membranes as functional receptors, we measured the macroscopic GABA-evoked currents and macroscopic kinetic properties of wt and variant receptors (Fig. 4E and F). Remarkably, peak amplitudes of currents recorded from cells transfected with variant γ2(T90R) subunits were greatly decreased compared to those transfected with wt γ2 subunits (Fig, 4E) (*P *<* *0.0001, Table 1). In addition, the variant receptor currents had faster desensitization (*P *=* *0.0073, Table 1), increased desensitization extent (*P *<* *0.0001, Table 1) and slowed activation of the currents (*P *<* *0.0001, Table 1) without changes in deactivation (*P *=* *0.968, Table 1) (Fig. 4F).

#### The *de novo* variant γ2(T90R) subunit reduced substantial surface expression of GABA_A_ receptor α1, β3 and γ2(T90R) subunits

**Figure 7:**
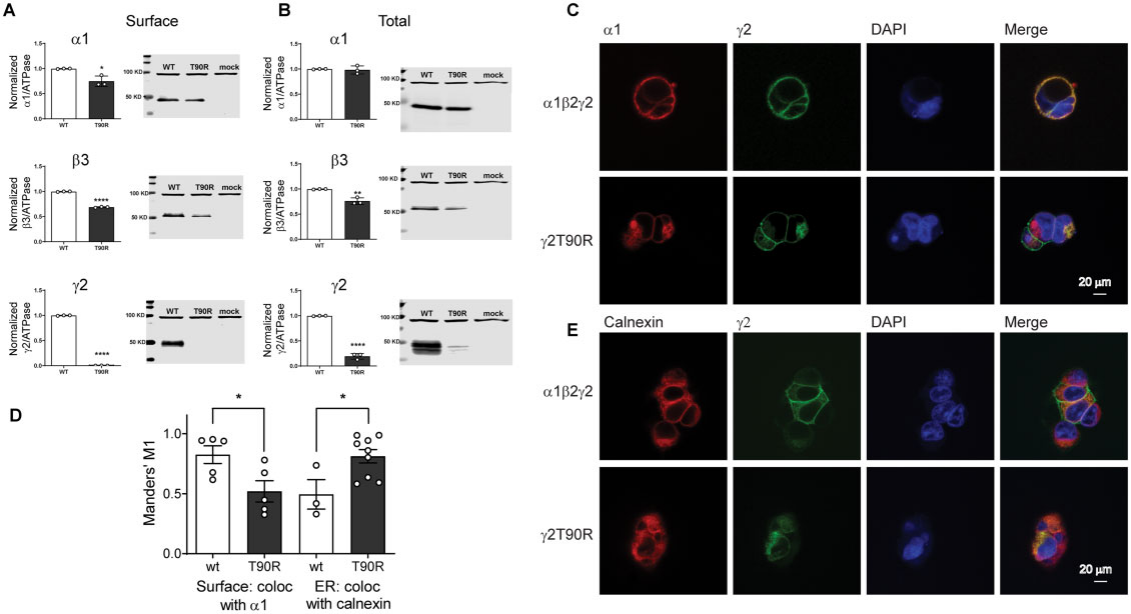
**The Dravet Syndrome *GABRG2* T90R variant is mainly retained in the ER.** Wt γ2 or variant γ2(T90R) subunits were coexpressed with α1 and β3 subunits in HEK293T cells. Surface (**A**) and total expression (**B**) were assessed as shown in Fig. 3. Values reported are mean ± SEM (Supplementary Table 3). Unpaired two-tailed Student's *t* test was used to determine significance relative to wild type (WT). ****P < 0.0001, **P < 0.01 and *P < 0.05, respectively. Corresponding uncropped blots are available in Supplementary data. (**C, E**) Confocal images of surface and intracellular immunofluorescence staining in HEK293 cells expressing α1β2γ2 wild type and variant γ2(T90R) receptors. Non-permeabilized cells were stained with antibodies against the α1 subunit (*red*) and the wt or variant γ2^HA^ tag (*green*). The ER was visualized with anticalnexin antibody (*red*). DAPI nuclear counterstaining (*blue*) and the merge of the staining are shown as indicated. (**D**) Quantification of the colocalization of variant γ2L(T90R)^HA^ subunits within the ER was measured using Manders’ coefficient M1. The Manders’ M1 indicated the fraction of γ2L subunits that colocalized on the surface or within the ER. Values reported are mean ± SEM (*n* = 3–9 experiments for each condition). Unpaired two-tailed Student's *t* test was used to determine significance relative to wild type (wt). *P < 0.05.

To assess surface trafficking of the variant γ2(T90R) subunits, we transfected HEK293T cells with α1, β3 and wt or variant γ2(T90R) subunits at a 1:1:1 α1:β2:γ2 subunit ratio and evaluated surface levels of wt and variant γ2 subunits by surface biotinylation (Fig. 7A). Compared to coexpressed wt γ2 subunits, we found that surface levels of coexpressed variant γ2(T90R) subunits were reduced substantially (*P *<* *0.0001, Supplementary Table 3). To further investigate whether the variant γ2(T90R) subunits had a dominant negative effect to decrease the trafficking of partnering subunits to the cell surface, we coexpressed α1 and β3 subunits with wt or variant γ2(T90R) subunits and analysed the surface levels of α1, β3 and γ2 or γ2(T90R) subunits (Fig. 7A). We confirmed that the surface levels of α1 (*P *=* *0.0148, Supplementary Table 3) and β3 (*P *<* *0.0001, Supplementary Table 3) subunits were significantly reduced in the presence of variant γ2(T90R) subunit. Moreover, total levels of variant γ2(T90R) (*P *<* *0.0001, Supplementary Table 3) and β3 subunits (*P *=* *0.0034, Supplementary Table 3) were significantly reduced (Fig. 7B). In contrast, the total amount of wt α1 subunits was not altered (*P *=* *0.752, Supplementary Table 3). While the significant reduction of the surface of α, β and γ subunits confirmed the major reduction of GABA-evoked currents, the total reduction of solely β and γ subunits suggested disruption of the assembly and trafficking of receptors, due to inefficient receptor assembly and trapping of partnering subunits in the ER hindering their assembly and trafficking.

#### The *de novo* variant γ2(T90R) subunit had different surface and intracellular distributions than wild-type subunits

Because the variant γ2(T90R) subunits had different total and surface expression levels, we extended our study to determine and compare the cellular locations of variant and wt γ2 subunits in HEK293T cells using confocal microscopy (Fig. 7C–E). Transfected cells coexpressing wt α1 and β2 subunits with wt γ2 L^HA^ or variant γ2 L^HA^(T90R) subunits were fixed and stained with anti-α1 subunit (red) and anti-HA (green) antibodies. Without cell permeabilization, the receptors at the cell surface were labelled and surface expression and localization of γ2 L^HA^ subunits and α1 subunits were determined.

Wt γ2 L^HA^ subunit signals were present on the surface and colocalized well with α1 subunit signals, consistent with coassembly of γ2 L^HA^ subunits with α1 and β2 subunits into receptors that were trafficked to the cell surface (Fig. 7C, yellow florescence is colocalization, top panels). In contrast, γ2 L(T90R)^HA^ had major reduction of surface HA signals (lack or reduction of yellow florescence in Fig. 7C, bottom panels).

Cells were then permeabilized and counterstained with antibodies raised against calnexin, an ER marker that shows a typical perinuclear and reticular distribution in the ER. While wt γ2 L^HA^ subunits were uniformly distributed intracellularly (Fig. 7E, top panels), variant γ2 L(T90R)^HA^ subunits intensely labelled an intracellular compartment consistent with the ER (Fig. 7E, bottom panels).

The interaction between wt γ2 L and variant γ2 L(T90R) subunits on the surface as measured by colocalization with the α1 subunit, and the ER by colocalization with calnexin was quantified using the MCC (Fig. 7D), which measures co-occurrence of two proteins independent of signal proportionality.^45^^,^^46^ Correlation between the signal intensities of wt γ2 and variant γ2(T90R) subunits with α1 subunits on the surface was significantly reduced for the variant subunit (wt 0.83 ± 0.07, *n* = 5; T90R 0.52 ± 0.09, *n* = 5, *P* = 0.0290). Further we observed that variant γ2 L(T90R) subunits had significantly increased colocalization with the ER (0.81 ± 0.06, *n* = 9, *P* = 0.0230), in comparison with wt γ2 subunits (0.50 ± 0.12, *n* = 3) (Fig. 7D).

## Discussion

### Mutations in multiple genes, (*GABRA1, GABRB2* and *GABRG2*), have a common target (α1β2γ2 GABA_A_ receptors) to cause Dravet syndrome

GABA_A_ receptors are important neurotransmitter receptors that control neuronal excitability. It is well known that a functional defect in these receptors causes a significant imbalance of neuronal excitation and inhibition that leads to disinhibition and hyperexcitability in the brain.^10^ Mutations in *GABRs* are associated with a wide spectrum of epilepsy syndromes from relatively benign inherited epilepsies (GEFS+, CAE, febrile seizures) to more catastrophic developmental EE syndromes (Dravet syndrome, infantile spasms, Lennox-Gastaut syndrome).^11–14^ Among the common *GABR* genes with widespread distribution in the CNS and association with inherited epilepsy syndromes are *GABRA1*, *GABRB2* and *GABRG2*. In addition, the rapid advances in NGS applied to cases with severe EEs has brought attention to the discoveries of additional variants and *de novo* mutations/variants in *GABRA1*,^6^^,^^7^^,^^9^^,^^47^ *GABRA2*,^48^ *GABRA3*,^49^ *GABRA5*,^47^^,^^48^ *GABRB^1^*,^50^ *GABRB2*,^23^^,^^51^^,^^76^ *GABRB3*^8^^,^^50^^,^^52^ and *GABRG2*^39^^,^^53^

In this study, we identified nine patients with Dravet syndrome caused by variants in three relevant, but different, genes, *GABRA1*, *GABRB2* or *GABRG2*. It is interesting that the GABA_A_ receptor subunits encoded by the genes in this study were the α1, β2 and γ2 subunits, which coassemble to form the α1β2γ2 receptor, the most common GABA_A_ receptor in the CNS. The α1β2γ2 receptor is abundant and comprises about half of all GABA_A_ receptors.^54^ They are widely distributed in the CNS, especially on neocortical and hippocampal interneurons, and so individuals with any one of the Dravet syndrome associated *GABRA1*, *GABRB2* or *GABRG2* variants would have widespread impairment of α1β2γ2 receptors in the CNS despite having the variants in one of three different *GABR genes*. The finding that variants in each of these three different subunit genes all produce Dravet syndrome suggests that they all primarily reduce function of the same α1β2γ2 receptors throughout the CNS. In general, the Dravet syndrome variants we are reporting were located in structural domains closely related to the GABA binding site or the pore domain of the channel. Thus, regardless of the GABA_A_ receptor subunit subtype that carried the mutation/variant, the assembled receptor ended up with defective expression or function, which was determined by the location of the mutation/variant in the well-known structural motifs that define the gating/conductance^55–58^ or assembly/trafficking^41–44^ domains of GABA_A_ receptor channels.

### *GABRA1* and *GABRB2* Dravet syndrome variants that decreased gating

The α1(R214C), α1(L215P) and β2(Y181F) subunit variants were all located in the β/α interface in the GABA-binding domain. The two α1 subunit variants reduced peak α1β2γ2 receptor current amplitudes by 60% but did not alter surface expression of α1, β2 or γ2 subunits. In contrast, the β2(Y181F) subunit variant did not alter substantially peak GABA_A_ receptor currents and slightly increased surface expression of the variant β2 subunits. However, all these variants produced a major acceleration of macroscopic deactivation of the receptor. Previous studies attributed this phenomenon to the destabilization of the liganded open state of the receptor due to the loss of affinity for the agonist.^58^ Mutagenesis studies identified the group of residues within the P202-D219 segment of the α1 subunit that were part of the GABA binding pocket^59^ and were the dynamic component during channel activation transitions. In addition, previous studies reported that Y181 of the β2 subunit was required for GABA-dependent activation.^60^ In fact, the recent α1β3γ2 structure revealed that the orthosteric ligand binding site for GABA is within an ‘aromatic box’ that includes Y181.^26^ At GABAergic synapses, inhibitory postsynaptic current (IPSCs) decay is shaped primarily by intrinsic GABA_A_ receptor kinetic properties.^61^^,^^62^ The functional implication of faster deactivation of GABA-evoked variant currents is that this is a mechanism for shortening individual IPSCs that develops over time, thereby decreasing functional inhibition at high activation frequencies and resulting in hyperexcitability.^63^^,^^64^

### α1 and β2 subunit Dravet syndrome variants that removed desensitization–deactivation coupling

α1(V287I) and β2(F331del) subunit variants were located in the pore domain of the receptor. The α1(V287I) variant behaved similar to the α1(R214C) and α1(L215P) variants by reducing peak current amplitudes ∼60% without altering surface subunit expression. The β2(F331del) subunit variant had little effect on current amplitude or surface expression levels. Moreover, neither of the two variant α1(V287I) and β2(F331del) subunits altered activation of the receptor. However, the desensitization and deactivation kinetics of the variant currents seemed uncoupled.^58^ Typically, desensitization and deactivation of GABA_A_ receptor currents are ‘coupled’; if desensitization is accelerated, deactivation slows and vice versa. However, despite desensitization of both variant currents being prolonged, current deactivation did not accelerate as expected if there was desensitization-deactivation coupling.^56^ Thus, the β2(F331del) subunit variant also prolonged current deactivation, but the α1(V287I) subunit variant did not affect it. These findings are consistent with the notion that the receptor desensitization gate is a functional and structural entity that is different from the activation gate.^31^ Decreased desensitization may be caused by decreased desensitized state occupancy or increased open state occupancy.^65^ The later could not be the case since both variants had reduced peak currents. The macroscopic desensitization of GABA_A_ receptor currents regulates the duration of IPSCs,^66^ which ultimately shapes the GABAergic input of inhibitory circuits. Previous studies suggested that the desensitized states represent alternative receptor conformations with high affinity for the agonist, which prolongs the time liganded receptors reopened.^66^ However, the variant receptors seemed to favour a non-conducting liganded state with late entries into open states. The recent solved structure of the GABA_A_ receptor confirmed that the desensitization gate of the receptor is in the pore domain,^31^ where the most distal segments of the M2 and M3 helices of adjacent subunits are in contact with the intracellular face. Interestingly, the α1V287I and β2(F331del) subunit variants were found in this structural belt that delineates the receptor desensitization gate.^57^ The α1(V287I) subunit variant is at the 5ʹ position of M2 in the pore, which is right above the constriction of the pore at its cytoplasmic end, between the -3′ and 4′ M2 positions. Mutations at the M2 and M3 interface of adjacent α and β subunits between -3′ and 4′ positions strongly affected desensitization without altering activation gating efficacy.^57^

The residues at the intracellular end of M3 that are part of the interaction network around the desensitization gate are also part of the PIP_2_ interaction network revealed in GABA_A_ receptors.^31^ In general, it is known that PIP_2_ regulates the gating of ion channels by binding to cationic clusters found at the interface of the transmembrane helices and cytoplasmic regions.^67^^,^^68^ The discovery of PIP_2_ binding pockets at the interfaces of α1 subunits revealed that GABA_A_ receptors are not an exception to this modulation. We found that the β2(F331del) subunit predicted a shortening of the end of the M3 helix exposing positive charged residues at the cytoplasmic interface homologous to the PIP_2_ binding site. Although it is predicted that the variant favours a secondary site for PIP_2_ binding, the mechanism is unclear but leads to speculation that this could cause allosteric conformational changes in the desensitization gate, which could account for impairing desensitization–deactivation coupling of currents in receptors carrying the variant. Previous studies in pentameric ligand-gated ion channels showed that the direct binding of anionic phospholipids at the interfacial regions of the TM reduces channel desensitization by stabilizing the open state, while perturbations of the lipid-binding site accelerate desensitization.^69^^,^^70^ The cryo-EM structure of the GABA_A_ receptor revealed PIP_2_ bound to the M1–M2 loop, post-M3 and pre-M4 segments of α1 subunits.^31^ Depletion of PIP_2_ by co-application of etomidate and poly-L-lysine to inside-out patches seemed to enhance etomidate-evoked currents. Although it is unclear whether allosteric activation of the receptor modulates differently the desensitization gate and the binding of PIP_2_, these observations do not rule out the possibility of the receptor stabilization in a different conformational state that affects both desensitization gate and binding sites of PIP_2_.

### The β2(F331S) variant

Unlike the Dravet syndrome-associated variants in the α1, β2 and γ2 subunits, the variant β2(F331S) subunit did not affect peak GABA-evoked current amplitudes and had no apparent effects on the kinetics of the receptor. In contrast, the β2(F331S) variant subunits increased surface β2 subunit levels, without altering α1 or γ2 subunit levels. The β2 subunit variant F331S was mapped at the edge of the M3 helix in the N-terminus of the intracellular M3–M4 loop of the receptor, where this Phe is highly conserved across all GABA_A_ receptor β subunits. Despite the fact that it is not clear what the result of an increase of β subunits on the cell surface would be, several studies indicated the importance of GABA_A_ receptor associated proteins in trafficking and internalization of receptors through interactions at the intracellular M3–M4 loop.^71–73^ It is noteworthy that BIG2, a 200‐kDa protein belonging to a class of high molecular weight GDP/GTP exchange factors that catalyzes GDP/GTP exchange on the small G‐protein ADP‐ribosylation factors,^74^ was reported to interact with a stretch of residues at the edge of the M3-helix of the β subunit,^75^ where F331 is located. It seemed that BIG2 facilitated the exit of GABA_A_ receptor subunits from the ER, and then enhanced the trafficking of β subunits to the surface.^75^ This may be the mechanism behind the slight increase in β2(F331S) variant subunits in the membrane. More importantly, BIG2 is present at GABAergic inhibitory synapses where it is colocalized with GABA_A_ receptors.^75^ This might indicate a regulation of the neural excitability of the circuits containing this variant, and perhaps a mechanism of hyperexcitability leading to Dravet syndrome.

### The Dravet syndrome variant that is trafficking deficient

The γ*2*(T90R) subunit variant is found at the γ2+/β2-subunit interface, a region that contains required structural motifs for proper folding and assembly of GABA_A_ receptors.^41–44^ Mutations in this region resulted in intracellular retention and reduced surface expression of GABA_A_ receptors.^21^ The γ*2*(T90R) subunit variant substantially reduced peak current amplitudes by 90% and altered current kinetic properties. Moreover, the γ*2*(T90R) subunit variant produced a large reduction of surface γ2 subunit expression and minimal to no reduction of surface expression of α1 and β3 subunits, suggesting that T90R reduced γ2 subunit biogenesis, leaving the assembly of α1β2 receptors. We found a second case harbouring the *de novo* γ2(P342L) subunit variant with the same epilepsy phenotype. The γ2(P342L) subunit variant corresponds to the γ2(P302L) subunit variant,^39^ which indicated that the cytoplasmic channel pore domain of the receptor where the inactivation gate resides is a ‘hot spot’ for disease-causing variants. As we reported previously, the γ2(P302L) subunit variant produced a small reduction of surface expression of α1, β2 and γ2 subunits, but primarily reduced ion channel function by producing increased stability of the inactivation gate.^39^

### Severity of variants in *GABRA1, GABRB2, GABRB3* and *GABRG2* determine the nature of the seizure semiologies of each family of genetic epilepsy syndromes.

It is well known that mutations/variants in *GABRA1*, *GABRB2, GABRB3,* or *GABRG2* produce several different types of epilepsy.^13^^,^^14^ There are variants in *GABRA1*, *GABRB2* or *GABRG2* that are all associated with Dravet syndrome. There are also variants in *GABRA1*, *GABRB3* or *GABRG2* that are all associated with GGEs ranging from CAE, generalized epilepsy with febrile seizures plus (GEFS+), myoclonic atonic epilepsy (MAE) to other developmental EEs. All of these syndromes can be seen with variants in *GABRG2* as well as *GABRA1* and *GABRB2,3*.

In contrast, there are epilepsy syndromes associated with variants in *GABRA1* and *GABRB3* (IS), but not with *GABRG2*, with *GABRB3* (LGS) but not with *GABRA1* or *GABRG2* and with *GABRA1,2,5* and *GABRB2,3* [early onset EE (EOEE)] but not with *GABRG2*. This suggests that there are *GABRG2* epilepsy syndromes associated with *GABRG2* variants that may have the same receptor targets (α1, β2,3 or γ2 subunits or α1β3γ2 receptors) and non*GABRG2* epilepsy syndromes that have different non*GABRG2* targets (α1 or β2,3 subunits or α1β2,3 receptors). This also suggests that there are epilepsy syndromes associated with variants in α1β3γ2 receptors that contain γ2 subunits (Dravet syndrome, CAE, GEFS+, MAE) (γ2 subunit epilepsies) and other epilepsy syndromes associated α1β3 receptors that do not contain γ2 subunits (IS, LGS, EOEE, Juvenile myoclonic epilepsy) (nonγ2 subunit epilepsies).

Each of the variants discussed above has mild to severe epilepsy syndromes associated with it. How do individual variants in the same gene produce epilepsies with different seizure semiologies? Based on individual study of many human variants, it is likely that the ‘severity’ of the variant determines the epilepsy semiology. For example, the Dravet syndrome variants tend to be quite severe. Our data suggest that the α1, β2 and γ2 subunit variants associated with Dravet syndrome reported here impaired α1β3γ2 receptors differently. Variant α1 subunits decreased peak current and altered current kinetic properties without affecting surface trafficking, variant β2 subunits affected current kinetic properties but did not affect peak currents or surface expression, and mutant γ2 subunits decreased peak currents by impairing receptor trafficking or ion channel function. The effects of the variant on receptor dysfunction are likely due to the intrinsic properties of the subunit in the receptor. For example, for a variant to affect ligand binding, a subunit involved in GABA binding (α or β subunit) must be mutated. Remarkably, main association studies corroborate our hypothesis that missense variants, rather than nonsense variants, through a physio-pathological functional alteration of the protein, rather than by haploinsufficiency, are the main cause of the epilepsies.^13^^,^^14^ Moreover, the inhibitory *GABR*s were enriched for missense variants across developmental EEs and GGEs.

## Supplementary material

Supplementary material is available at *Brain Communications* online.

## Supplementary Material

fcab033_Supplementary_Data

## Abbreviations

AED =: antiepileptic drugs
CAE =: childhood absence epilepsy
EOEE =: early onset EE
EEs =: epileptic encephalopathies
GEFS+ =: genetic epilepsy with febrile seizures plus
GGEs =: genetic generalized epilepsies
GTCS =: generalized tonic-clonic seizures
gnomAD =: genome aggregation database
HS =: hemiclonic seizures
IS =: infantile spams
IPSCs =: inhibitory postsynaptic current
LGS =: Lenox-Gastaut syndrome
MCC =: Manders’ colocalization coefficient
MVD =: Molegro Virtual Docker
MAE =: myoclonic atonic epilepsy
NGS =: next-generation sequencing
PIP_2_ =: phosphatidylinositol-4,5-bisphosphate
RMSD =: root mean square deviation
SE =: status epilepticus
