# Correction to: Dravet syndrome-associated mutations in *GABRA1*, *GABRB2* and *GABRG2* define the genetic landscape of defects of GABA_A_ receptors

**DOI:** 10.1093/braincomms/fcac156

**Published:** 2022-06-24

This is a correction to: Ciria C. Hernandez, XiaoJuan Tian, Ningning Hu, Wangzhen Shen, Mackenzie A. Catron, Ying Yang, Jiaoyang Chen, Yuwu Jiang, Yuehua Zhang, Robert L. Macdonald, Dravet syndrome-associated mutations in *GABRA1*, *GABRB2* and *GABRG2* define the genetic landscape of defects of GABA_A_ receptors, *Brain Communications*, Volume 3, Issue 2, 2021, fcab033, https://doi.org/10.1093/braincomms/fcab033

In the originally published version of this manuscript, a reference was omitted. The authors have added a clarification that the three cases of *GABRB2* mentioned in the article have been reported previously. A reference to the previous report has been added.

