# Supplementary material for: Dravet syndrome-associated mutations in GABRA1, GABRB2 and GABRG2 define the genetic landscape of defects of GABAA receptors
Source: Brain Commun. 2021 Mar 11;3(2):fcab033. doi: 10.1093/braincomms/fcab033 (PMC8176149; doi:10.1093/braincomms/fcab033)

SUPPLEMENTARY DATA

DRAVET SYNDROME ASSOCIATED MUTATIONS IN *GABRA1*, *GABRB2* AND *GABRG2* DEFINE THE GENETIC LANDSCAPE OF DEFECTS OF $GABA_A$ RECEPTORS.

Ciria C. Hernandez#,[1, 2] XiaoJuan Tian#,[3,4] Ningning Hu,[2] Wangzhen Shen,[2] Mackenzie A. Catron,[2, 5] Ying Yang,[3] Jiaoyang Chen,[3] Yuwu Jiang,[3,6] Yuehua Zhang,*[3] and Robert L. Macdonald.*[2]

[1]Life Sciences Institute, University of Michigan, Ann Arbor, MI 48198, USA.

[2]Department of Neurology, Vanderbilt University, Nashville, TN 37240, USA.

[3]Department of Pediatrics and Pediatric Epilepsy Center, Peking University First Hospital, Beijing 100034, China.

[4]Department of Neurology, Beijing Children's Hospital, Capital Medical University, National Center for Children's Health, Beijing 100045, China.

[5]Neuroscience Graduate Program, Vanderbilt University, Nashville, TN 37240, USA.

[6]Center of Epilepsy, Beijing Institute for Brain Disorders, Beijing 100069, China.

Supplementary Table 1. Genetic and Clinical Features of Dravet Syndrome patients carrying GABRA1 variants identified in this study.				
GABRA1				
	**Patient 1**	**Patient 2**	**Patient 3**	**Patient 4**
**Inheritance**	De Novo	De Novo	De Novo	De Novo
**Genomic position***	chr5-161309648	chr5-161309644	chr5-161322674	chr5-161309645
**Nucleotide change**	c.644T>C	c.640C>T	c.859G>A	c.641G>A
**Amino acid change**	L215P	R214C	V287I	R214H
**Sex**	M	F	M	M
**Current age**	12y	11y	9y	5y
**Age at onset**	4m	8m	6m	6.5m
**Seizure type at onset**	Febrile GTCS 3-4 min	Hot shower 25 min	Hot shower 3-5 min, and post-vaccination	Febrile, hot shower 3 min
**Fever-sensitive**	Yes	Yes	Yes	Yes
**Light-sensitive**	Yes	No	No	No
**Seizure types**	GTCS, HS, MS, SE	Focal, HS, MS, SE	GTCS, HS, ATAS, SE, photosensitivity	GTCS, HS, focal, SE
**AED response**	VPA+TPM+CLB	VPA+LEV, OXC exacerbated	VPA+LEV, OXC exacerbated	VPA+LEV+LTG, OXC exacerbated

**Seizure outcome**	Weekly	Yearly, Seizure free for 6 y. Relapse after AED withdrawal	Yearly	Monthly
**EEG**	Generalized spike-wave complex and poly-spike-wave complex in the occipital area at the awake period. Frequent myoclonic seizures were monitored at the awake period and IPS, with a quick shake of the head or the body. (7y)	Normal (9m); Atypical low-amplitude sharp wave were monitored sometimes at left central and parietal area in the sleep period. (2y 5m)	Each lead 2-4Hz slow waves were paroxysmal monitored, with few sharp-waves. (7 m)	Normal (6.5m, 7m); Atypical spikes in the right anterior or bilateral temporal region during sleep(11m); Normal(1y9m); Atypical spikes in bilateral anterior head during sleep (2y 5m).
**Motor development**	Normal	Normal	Mild delay	Normal
**Cognitive outcome**	Mild delay	Mild delay	Mild delay	Mild delay
**MRI findings**	Normal (7y)	Normal (2y 5m)	Normal (7m)	Subarachnoid width in the frontotemporal area(2y5m)
[1]**Ensembl report genetic variation**	NR (L215V; rs796052490)	rs727503940	rs796052493 (V287L; rs796052493)	rs886039373

SE, status epilepticus; AEDs, antiepileptic drugs; GTCS, generalized tonic-clonic seizure; HS, hemiclonic seizure; ATAS, atypical absence seizure; MS, myoclonic seizure; VPA, valproate acid; TPM, topiramate; LEV, levetiracetam; OXC, oxcarbazepine; CZP, clonazepam; LTG, lamotrigine; CLB, clobazam; VitB, Vitamin B6 or pyridoxine; Sz, seizure; FS, febrile seizures. Genomic positions are related to the GRCh37/hg19 human genome assembly. [1]Ensembl GRCh37 release 97 - July 2019 © EMBL-EBI. NR, non-reported.

Supplementary Table 2. Genetic and Clinical Features of Dravet Syndrome patients carrying GABRB2 and GABRG2 variants identified in this study.

	GABRB2			***GABRG2***	
	**Patient 5**	**Patient 6**	**Patient 7**	**Patient 8**	**Patient 9**
**Inheritance**	De Novo	AD, Maternal	De Novo	De Novo	De Novo
**Genomic position***	chr5-160757975	chr5-160757975-160757977	chr5-160763776	chr5:161569305	chr5-161522510
**Nucleotide change**	c.992T>C	c.990_992del	c.542A>T	c.1025C>T	c.269C>G
**Amino acid change**	F331S	F331del	Y181F	P342L	T90R
**Sex**	M	F	M	M	F
**Current age**	7y	12y	3.5y	10.5y	16y
**Age at onset**	6m	8m	6.5m	7m	7m
**Seizure type at onset**	Febrile focal seizure, 15 min	Febrile GTCS, 1 min	Febrile GTCS, 1 min	Febrile GTCS, 2 min	Febrile hot shower, 1 min
**Fever-sensitive**	Yes	Yes	Yes	Yes	Yes

**Light-sensitive**	No	No	No	No	No
**Seizure types**	GTCS, HS, focal, MS, SE	GTCS, HS, focal, MS, SE	GTCS, HS, focal, MS, SE	GTCS, HS, ATAS, MS, SE	GTCS, HS, ATAS, MS, SE
**AED response**	VPA+LEV	VPA+LEV	VPA+CZP+TPM OXC exacerbated	VPA+CZP, OXC exacerbated	VPA+TPM+LEV
**Seizure outcome**	Yearly	Yearly, seizure free for 6y6m	Monthly	Weekly	Yearly
**EEG**	Mass spike-wave and spike-wave complex charges were monitored at the right central parietal and middle-post temporal area, especially in the sleep period. (7y)	Normal (1y 3m; 2y); Higher amplitude spike and spike-slow waves (10y); Medium amplitude sharp and sharp-slow waves. (11y)	Normal (10m)	Normal (1y; 1.5y); Generalized 2-3Hz spike-wave complex, polyspike-wave complex charge. Many episodes of atypical absence seizures were monitored. (4y); Slow in background activity, generalized 2-4Hz spike-wave, multiple spike-wave charge. One suspected atypical absence seizures was recorded in awake period. (5y,3m)	In the interictal period bilateral multifocal spike-wave or spike-wave complex charge. (3y,6m); Sharp-wave complex or Sharp-wave complex with θ rhythm charge in frontal area, right mainly. (12y)
**Motor development**	Normal	Mild delay	Mild delay	Mild delay	Mild delay
**Cognitive outcome**	Mild delay	Mild delay	Mild delay	Moderate delay	Wechsler intelligence test (12y) :57

**MRI findings**	Normal (7y)	Normal (2y)	Normal (10m)	Normal (1.5y)	Bilateral ventricle enlarged, right hippocampal sclerosis was smaller than left with abnormal signals, right hippocampal sclerosis (4y6m)
**[1]Ensembl report genetic variation**	NR	NR	NR	NR	NR (T90M; rs1057520498)

SE, status epilepticus; AEDs, antiepileptic drugs; GTCS, generalized tonic-clonic seizure; HS, hemiclonic seizure; ATAS, atypical absence seizure; MS, myoclonic seizure; VPA, valproate acid; TPM, topiramate; LEV, levetiracetam; OXC, oxcarbazepine; CZP, clonazepam; LTG, lamotrigine; CLB, clobazam; VitB, Vitamin B6 or pyridoxine; Sz, seizure; FS, febrile seizures. Genomic positions are related to the GRCh37/hg19 human genome assembly. [1]Ensembl GRCh37 release 97 - July 2019 © EMBL-EBI. NR, non-reported.

Supplementary Table S3. Surface and total expression of $GABA_A$ receptor variants.									
**Surface**									
GABRA1	**α1(n=3)**	**SEM**	***p*[a]**	**β3(n=3)**	**SEM**	***p*[a]**	**γ2(n=3)**	**SEM**	***p*[a]**
**WT**	0.9967	0.003333	-	0.9967	0.003333	-	0.9967	0.003333	-
**L215P**	0.9747	0.05075	0.9680	1.08	0.02261	0.2508	1.065	0.08623	0.8671
**R214C**	0.8417	0.03775	0.0835	0.9137	0.02826	0.2532	0.8927	0.09738	0.6790
**V287I**	1.056	0.05889	0.6590	1.019	0.05552	0.9293	0.9847	0.08574	0.9990
GABRB2	**α1(n=3)**	**SEM**	***p*[a]**	**β2(n=3)**	**SEM**	***p*[a]**	**γ2(n=3)**	**SEM**	***p*[a]**
**WT**	0.9967	0.003333	-	0.9967	0.003333	-	0.9967	0.003333	-
**Y182F**	1.056	0.01762	0.8583	1.085	0.0177	0.0056	0.95	0.07808	0.8918
**F331S**	1.038	0.04188	0.9414	1.058	0.01337	0.0356	1.003	0.05185	0.9996
**F331del**	0.9513	0.1229	0.9270	1.13	0.01683	0.0004	0.967	0.06712	0.9670
GABRG2	**α1(n=3)**	**SEM**	***p*[b]**	**β3(n=3)**	**SEM**	***p*[b]**	**γ2(n=3)**	**SEM**	***p*[b]**
**WT**	0.9967	0.003333	-	0.9967	0.003333	-	0.9967	0.003333	-
**T90R**	0.7463	0.06085	0.0148	0.687	0.005292	<0.0001	0.001667	0.000667	<0.0001

**Total**									
GABRA1	**α1(n=3)**	**SEM**	***p*[a]**	**β3(n=3)**	**SEM**	***p*[a]**	**γ2(n=3)**	**SEM**	***p*[a]**
**WT**	0.9967	0.003333	-	0.9967	0.003333	-	0.9967	0.003333	-
**L215P**	0.843	0.04158	0.4738	0.9963	0.04447	>0.9999	0.9013	0.05985	0.6923
**R214C**	0.7487	0.1035	0.1649	0.8133	0.0727	0.2992	0.7117	0.03722	0.0598
**V287I**	1.082	0.1273	0.8190	1.08	0.1324	0.7986	1.022	0.128	0.9888
GABRB2	**α1(n=3)**	**SEM**	***p*[a]**	**β2(n=3)**	**SEM**	***p*[a]**	**γ2(n=3)**	**SEM**	***p*[a]**
**WT**	0.9967	0.003333	-	0.9967	0.003333	-	0.9967	0.003333	-
**Y182F**	1.049	0.06319	0.8306	1.013	0.01553	0.9779	0.9887	0.05783	0.9977
**F331S**	1.016	0.05414	0.9874	1.008	0.03973	0.9929	0.9657	0.04872	0.8978
**F331del**	1.042	0.06815	0.8785	1.085	0.0596	0.2798	0.937	0.02052	0.5964
GABRG2	**α1(n=3)**	**SEM**	***p*[b]**	**β3(n=3)**	**SEM**	***p*[b]**	**γ2(n=3)**	**SEM**	***p*[b]**
**W**	0.9967	0.003333	-	0.9967	0.003333		0.9967	0.003333	-
**T90R**	0.9807	0.04719	0.7522	0.759	0.03811	0.0034	0.1923	0.03267	<0.0001

[a]One-way ANOVA with Dunnett's multiple comparisons test; [b]Unpaired two-tailed Student's t test.

SUPPLEMENTARY DATA: Uncropped blots figures 3, 6 and 7.

DRAVET SYNDROME ASSOCIATED MUTATIONS IN *GABRA1*, *GABRB2* AND *GABRG2* DEFINE THE GENETIC LANDSCAPE OF DEFECTS OF GABA$_A$ RECEPTORS.

Ciria C. Hernandez#,[1, 2] XiaoJuan Tian[#,3,4] Ningning Hu,[2] Wangzhen Shen,[2] Mackenzie A. Catron,[2, 5] Ying Yang,[3] Jiaoyang Chen,[3] Yuwu Jiang,[3,6] Yuehua Zhang,[*3] and Robert L. Macdonald.[*2]

[1]Life Sciences Institute, University of Michigan, Ann Arbor, MI 48198, USA.
[2]Department of Neurology, Vanderbilt University, Nashville, TN 37240, USA.
[3]Department of Pediatrics and Pediatric Epilepsy Center, Peking University First Hospital, Beijing 100034, China.
[4]Department of Neurology, Beijing Children's Hospital, Capital Medical University, National Center for Children's Health, Beijing 100045, China.
[5]Neuroscience Graduate Program, Vanderbilt University, Nashville, TN 37240, USA.
[6]Center of Epilepsy, Beijing Institute for Brain Disorders, Beijing 100069, China.

Figure 3A

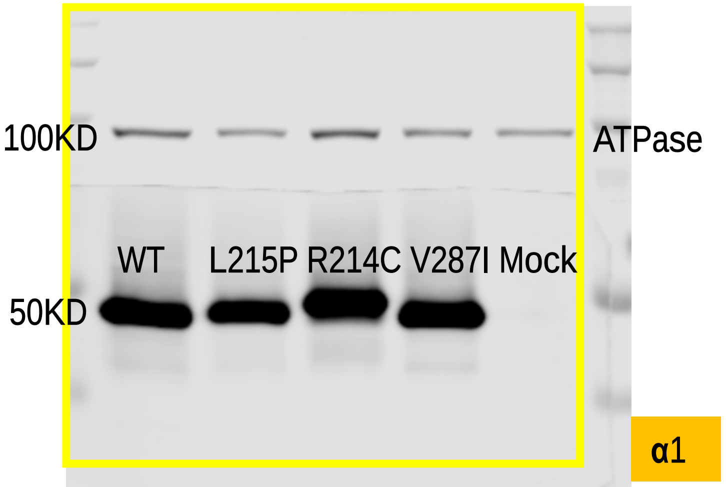

Figure 3B

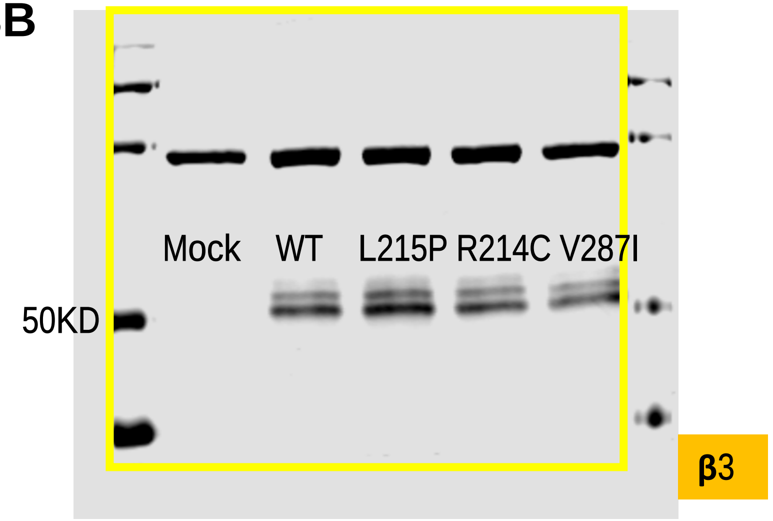

Figure 3C

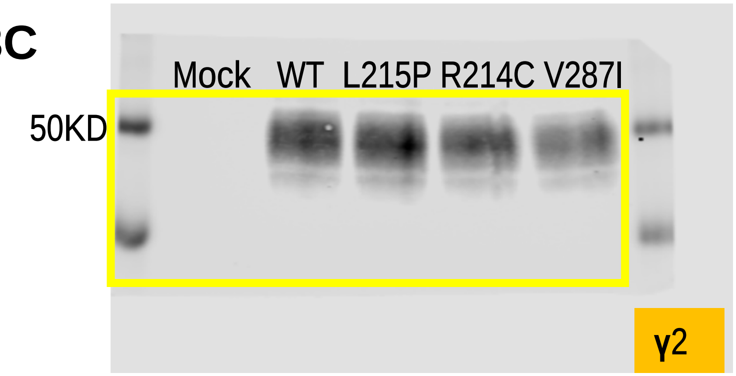

Figure 3-surface

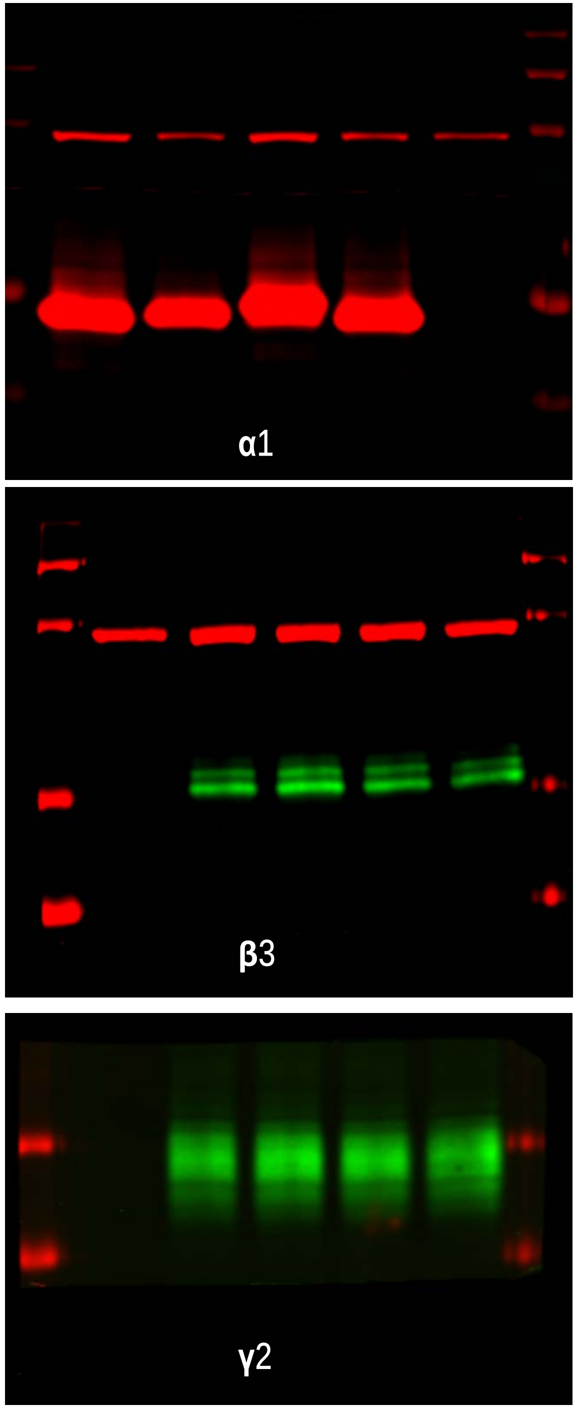

Figure 3D

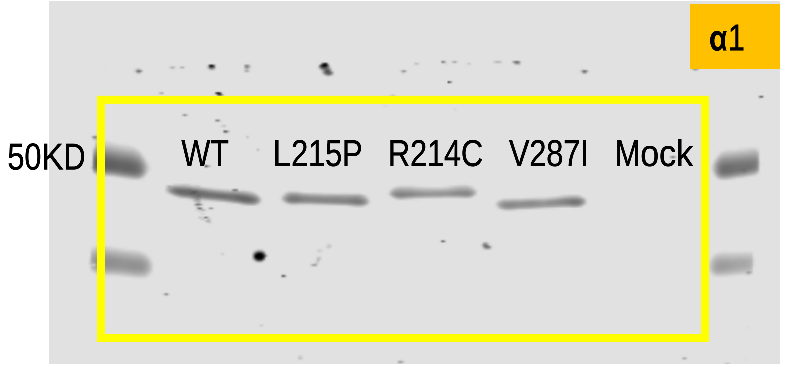

Figure 3E

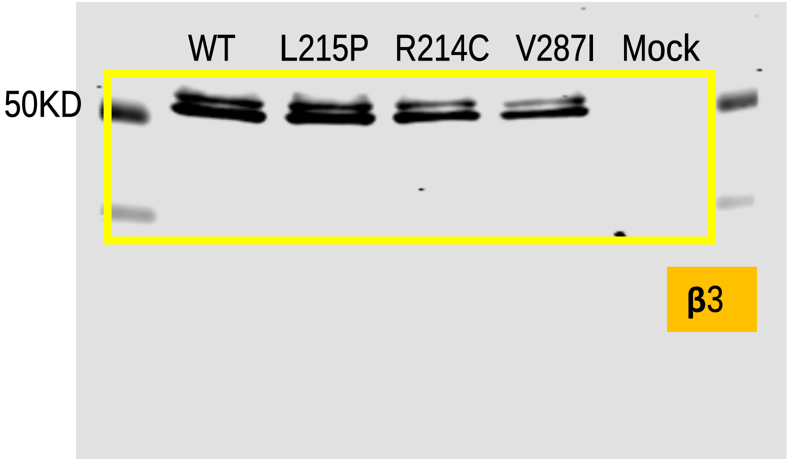

Figure 3F

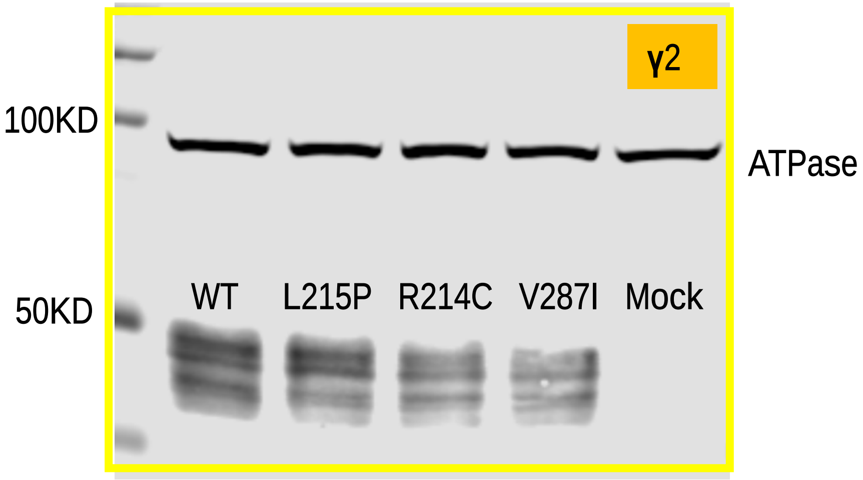

Figure 3-total

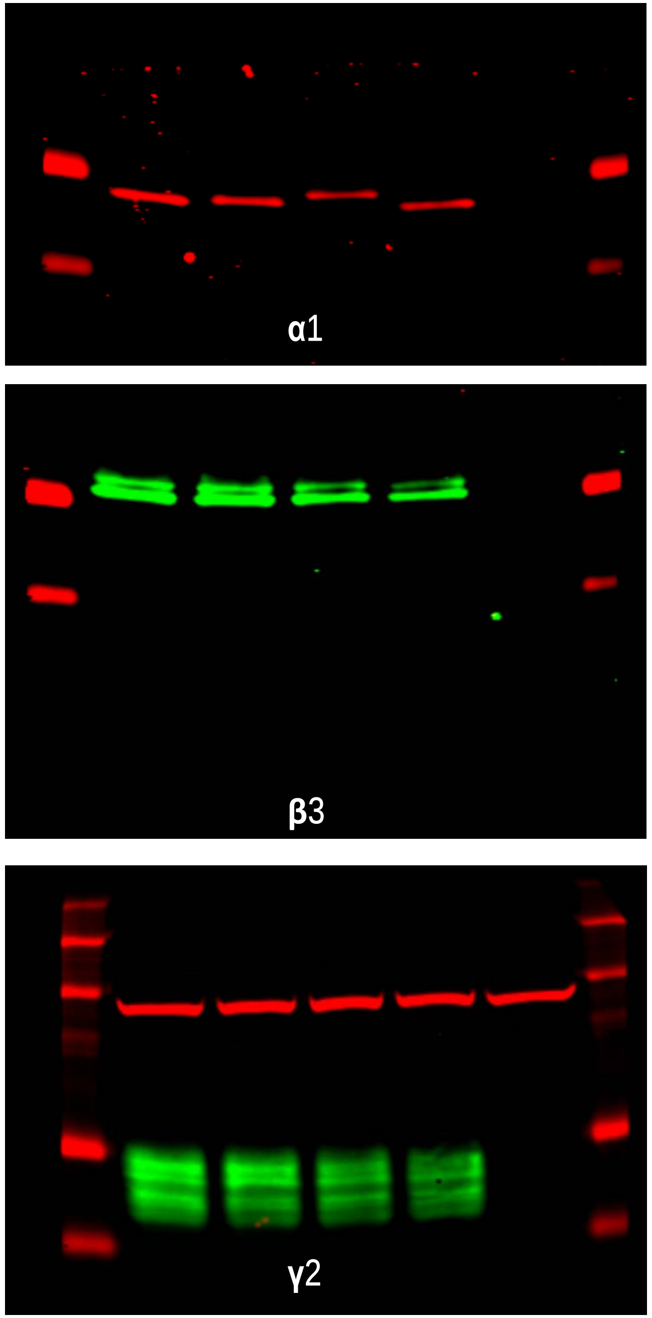

Figure 6A

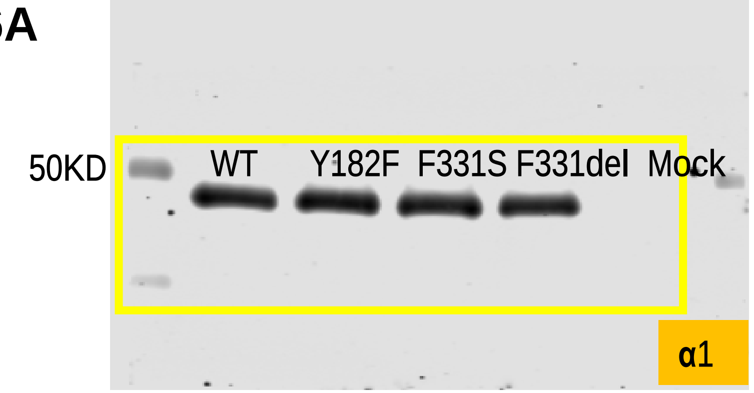

Figure 6B

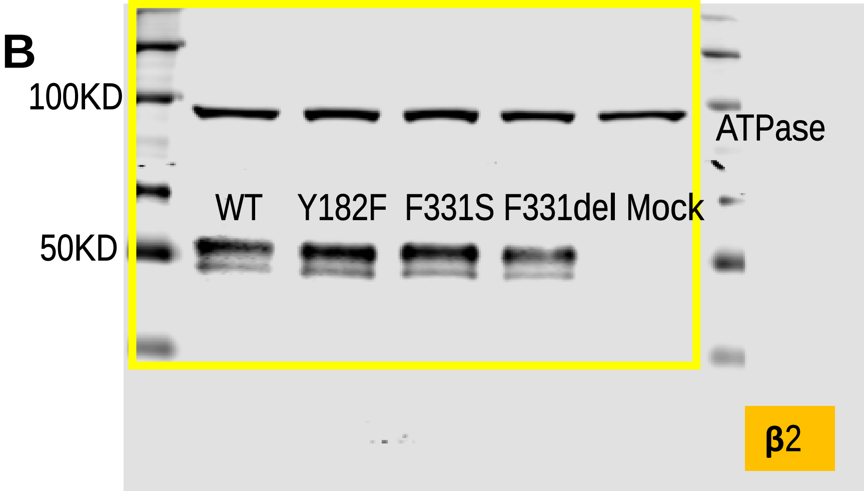

Figure 6C

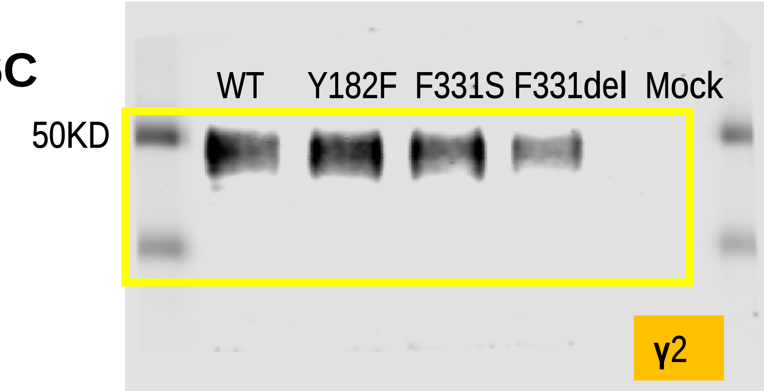

Figure 6-surface

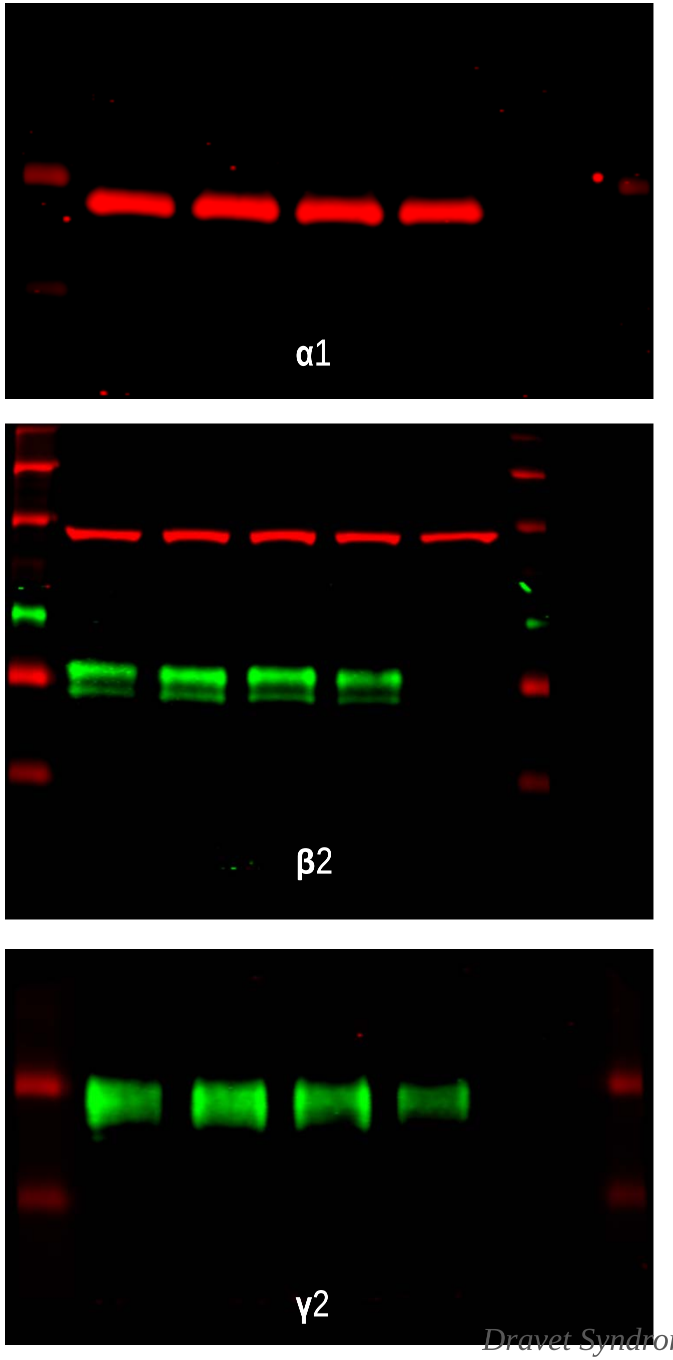

Figure 6D

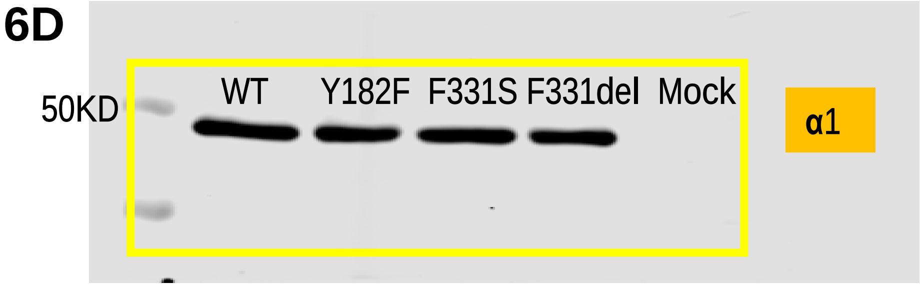

Figure 6E

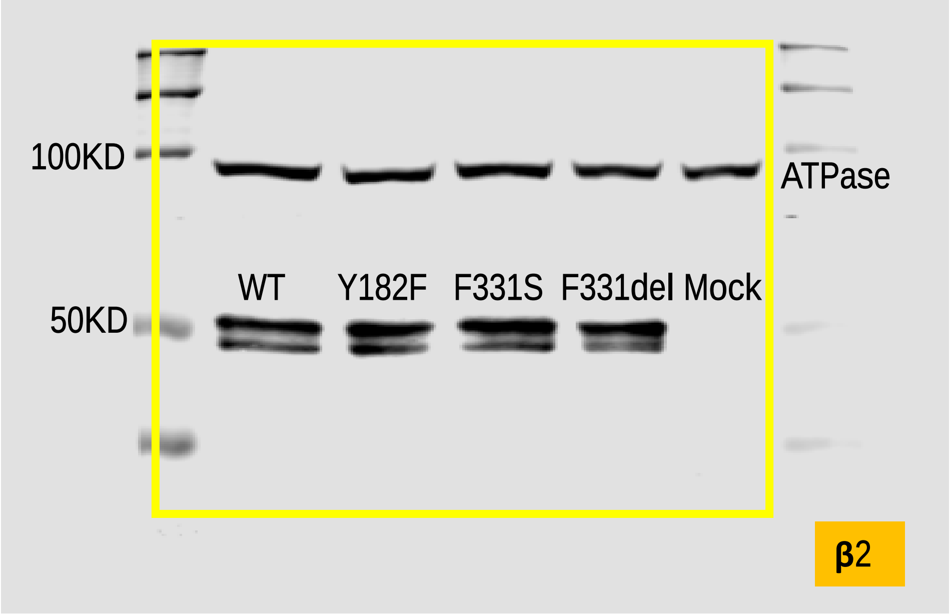

Figure 6F

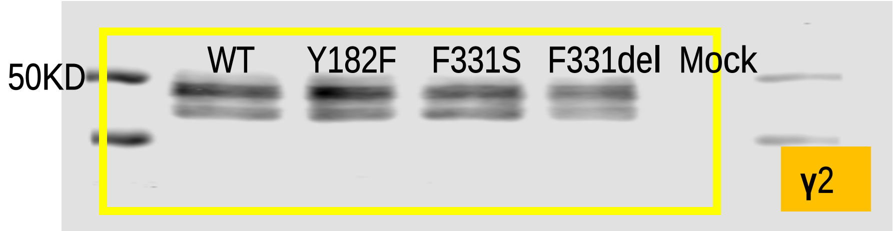

Figure 6-total

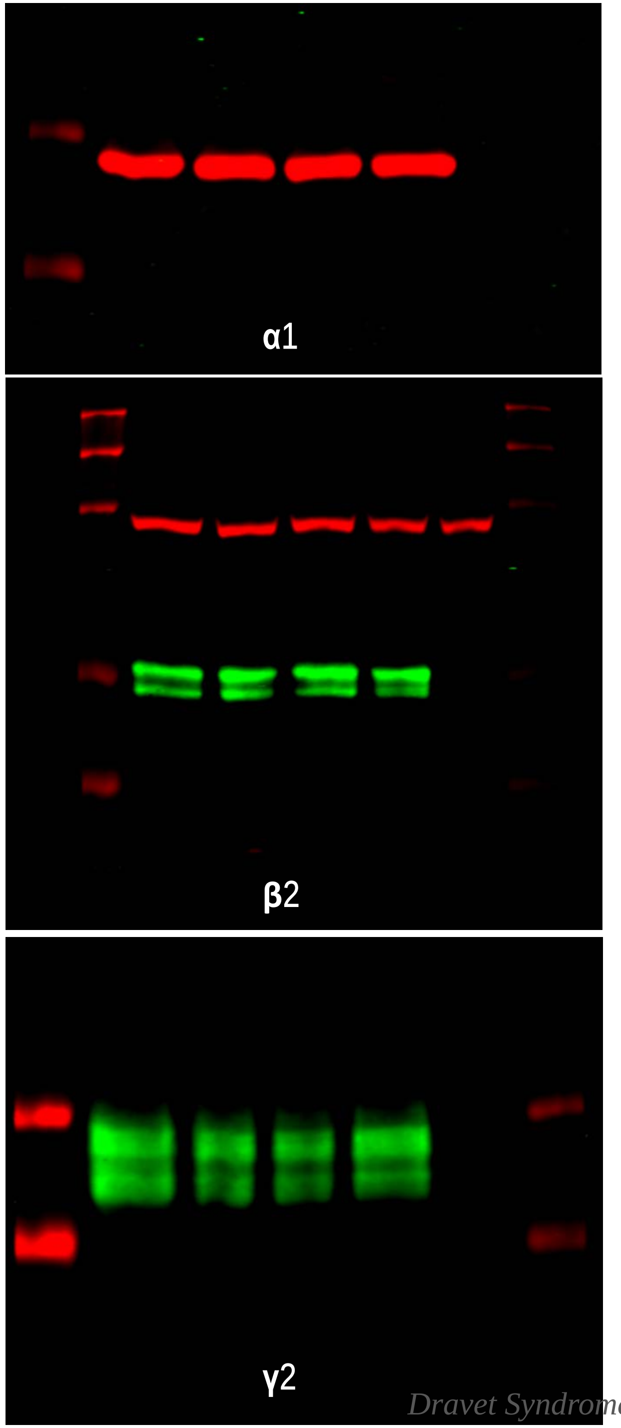

Figure 7-surface

Figure 7A, top panel

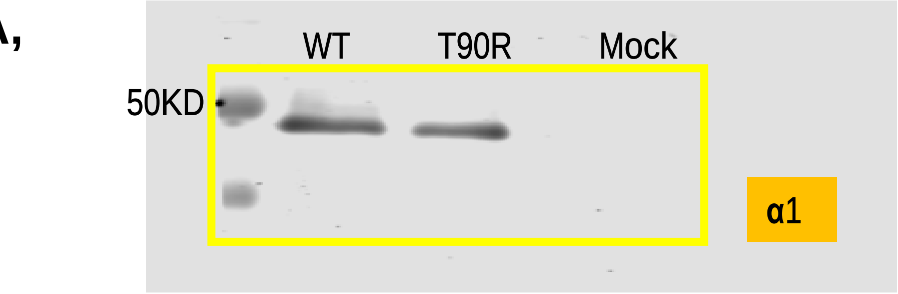

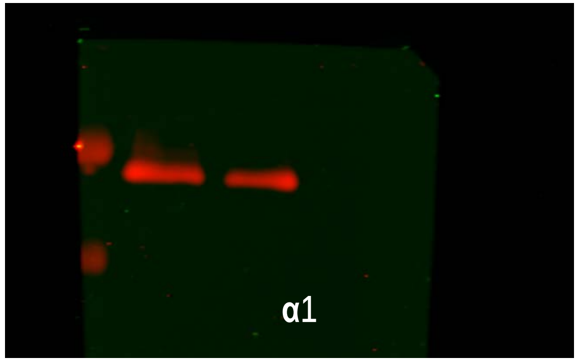

Figure 7A, middle panel

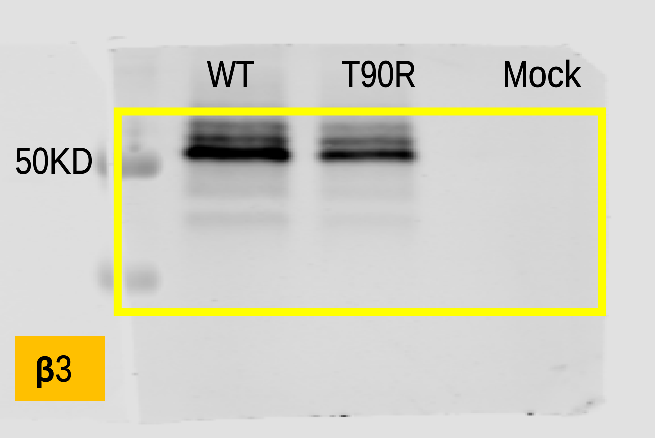

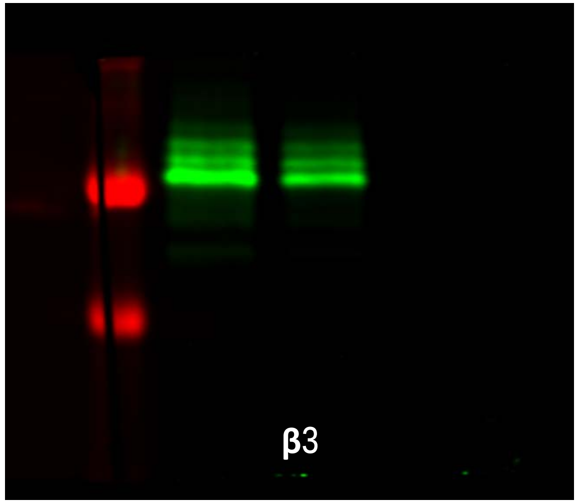

Figure 7A, bottom panel

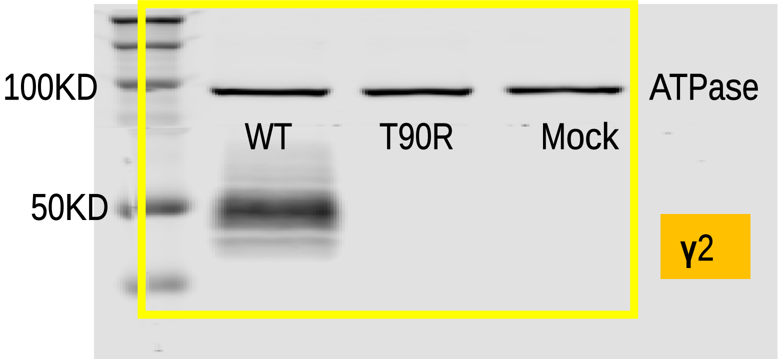

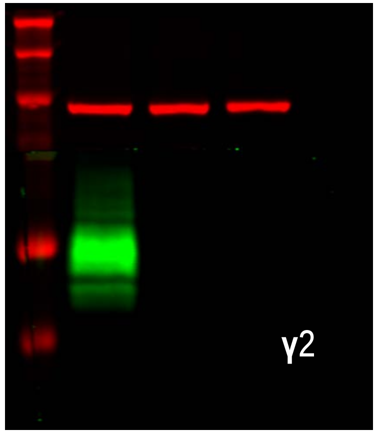

Figure 7-Total

Figure 7B, top panel

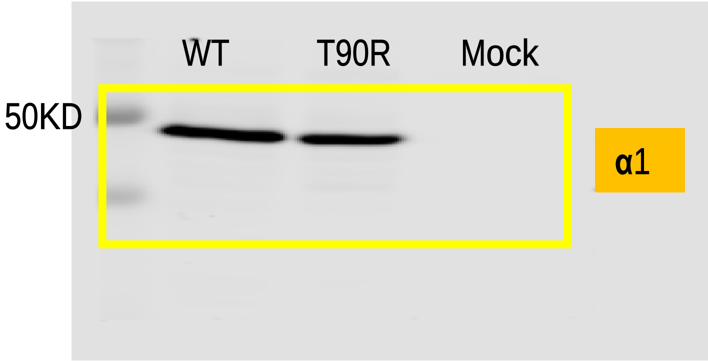

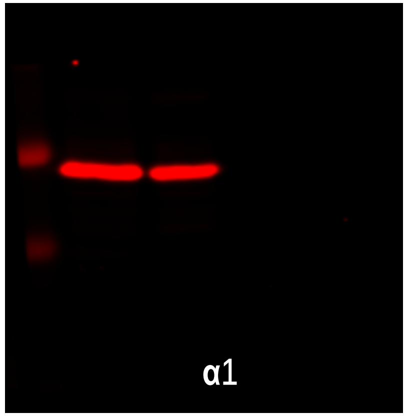

Figure 7B, middle panel

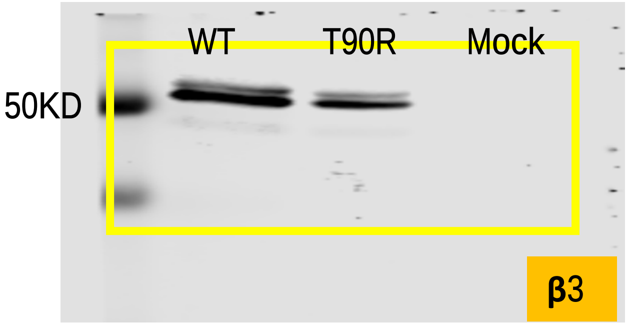

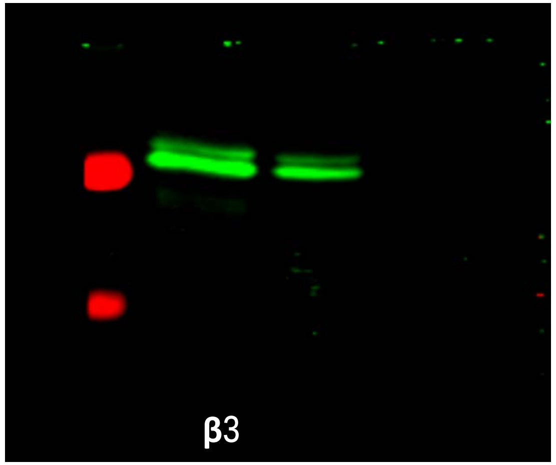

Figure 7B, bottom panel

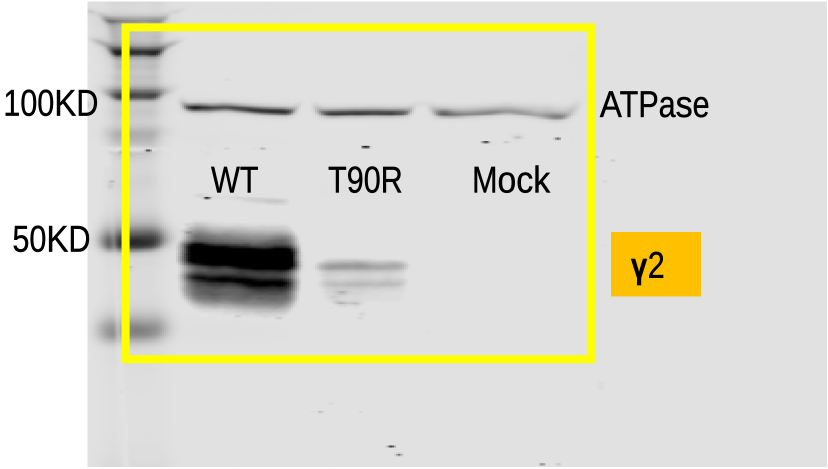

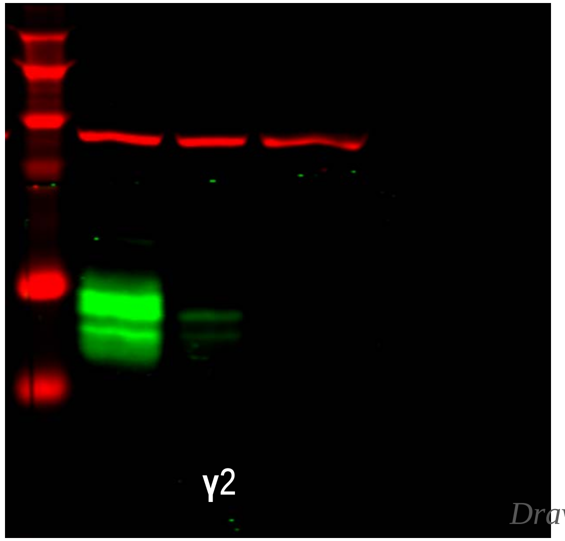

Supplement: fcab033_Supplementary_Data [file fcab033_Supplementary_Data.pdf]
